# The Extracellular Domains of IgG1 and T Cell-Derived IL-4/IL-13 Are Critical for the Polyclonal Memory IgE Response In Vivo

**DOI:** 10.1371/journal.pbio.1002290

**Published:** 2015-11-02

**Authors:** Adriana Turqueti-Neves, Manuel Otte, Christian Schwartz, Michaela Erika Renate Schmitt, Cornelia Lindner, Oliver Pabst, Philipp Yu, David Voehringer

**Affiliations:** 1 Department of Infection Biology, Institute for Clinical Microbiology, Immunology and Hygiene, University Hospital Erlangen and Friedrich-Alexander University Erlangen-Nuremberg, Erlangen, Germany; 2 Institute of Immunology, Hannover Medical School, Hannover, Germany; 3 Institute of Molecular Medicine, Medical Faculty, RWTH University, Aachen, Germany; 4 Institute for Immunology, Philipps-University Marburg, Marburg, Germany; Scripps Research Institute, UNITED STATES

## Abstract

IgE-mediated activation of mast cells and basophils contributes to protective immunity against helminths but also causes allergic responses. The development and persistence of IgE responses are poorly understood, which is in part due to the low number of IgE-producing cells. Here, we used next generation sequencing to uncover a striking overlap between the IgE and IgG1 repertoires in helminth-infected or OVA/alum-immunized wild-type BALB/c mice. The memory IgE response after secondary infection induced a strong increase of IgE^+^ plasma cells in spleen and lymph nodes. In contrast, germinal center B cells did not increase during secondary infection. Unexpectedly, the memory IgE response was lost in mice where the extracellular part of IgG1 had been replaced with IgE sequences. Adoptive transfer studies revealed that IgG1^+^ B cells were required and sufficient to constitute the memory IgE response in recipient mice. T cell-derived IL-4/IL-13 was required for the memory IgE response but not for expansion of B cells from memory mice. Together, our results reveal a close relationship between the IgE and IgG1 repertoires in vivo and demonstrate that the memory IgE response is mainly conserved at the level of memory IgG1^+^ B cells. Therefore, targeting the generation and survival of allergen-specific IgG1^+^ B cells could lead to development of new therapeutic strategies to treat chronic allergic disorders.

## Abstract 2

This study reveals that repertoires of IgE—the class of antibody that mediates allergic reactions—closely resemble those of IgG1, suggesting that the memory IgE response unfolds from IgG1-switched B cells (and not from IgM-expressing B cells) in response to T cell-derived cytokines.

## Author Summary

Allergic inflammation is initiated when IgE antibodies bind to high-affinity receptors on the cell surface of mast cells and basophils, thereby triggering the release of proinflammatory mediators. The development and persistence of IgE responses in vivo is poorly characterized because of the low number of IgE-producing B cells and plasma cells. Naïve mature B cells produce IgM antibodies. Upon activation, they “switch” class to produce IgG, IgA, or IgE antibodies. It is currently highly debated whether IgE-expressing B cells are generated by direct switching from IgM-expressing B cells or by sequential switching via IgG1-expressing B cells. Using next generation sequencing, we compared thousands of IgE, IgG1, and IgM sequences after immunization of mice with parasitic worms and found a striking overlap between the IgE and IgG1 repertoires. We further show that the memory IgE response to a secondary encounter with the same parasitic worms was dependent on T cell-derived cytokines. Genetically modified mice and adoptive transfers of B cells revealed that the memory IgE response is conserved at the level of IgG1-expressing B cells. These results favor the concept that bona fide IgE-expressing B cells do not exist, and memory IgE responses unfold from IgG1-expressing B cells, which undergo a secondary switch reaction and differentiation to plasma cells.

## Introduction

IgE probably emerged during mammalian evolution to defend hosts against parasites, since a correlation between high IgE levels and protection against helminths has been recognized [1,2]. Surprisingly, IgE was also found to mediate protection against bee venom in murine models [3,4]. However, IgE can also mediate adverse effects during allergic inflammation, leading in the most extreme case to death by anaphylaxis. Free IgE antibodies have a short half-life of only 12 h in the serum of healthy individuals [5]. IgE is by far the least abundant immunoglobulin isotype with about 10,000-fold lower serum concentrations as compared to IgM, IgG, or IgA isotypes. Class switch recombination (CSR) to IgE is induced by IL-4 or IL-13-mediated activation of STAT6. Activated STAT6 translocates to the nucleus, binds to the switch promoters in the Cε and Cγ1 genes, and in addition regulates expression of about 100 genes in B cells [6–8]. We recently discovered that STAT6 expression in B cells was required for germinal center (GC) formation in response to helminth infection and during allergic inflammation [9]. Infection of mice with the gastrointestinal helminth *Nippostrongylus brasiliensis* is a well-established model to study general mechanisms of IgE production in vivo. Serum IgE levels of *N*. *brasiliensis*-infected BALB/c mice increase up to 1,000-fold by day 14 after *N*. *brasiliensis* infection [10]. The IgE response to *N*. *brasiliensis* is abolished in IL-4-depleted [10] or IL-4-deficient [11] mice, indicating that IL-4 is the main cytokine that promotes IgE-CSR. However, IL-13 can also induce IgE-CSR under certain conditions, including the immune response to *Schistosoma mansoni* eggs [12,13]. IL-4 and/or IL-13 can be produced by many cell types of the adaptive and innate immune system such as Th2 cells, follicular T helper (T_FH_) cells, natural killer T (NKT) cells, basophils, eosinophils, mast cells, and type 2 innate lymphoid cells (ILC2). Although Th2 cells and T_FH_ cells are generally considered to be the most relevant cell types for induction of IgE-CSR in B cells, it remains unclear to what extent innate IL-4/IL-13-expressing cell types contribute to this process, especially during secondary infection when basophils and mast cells can be rapidly activated to release large amounts of IL-4/IL-13.

Immunohistological stainings indicated that IgE-CSR occurs outside GCs [14], while other studies identified IgE^+^ B cells inside GCs by using fluorescent IgE reporter mice [15–17]. Reporter mice are valuable tools, but they also bear certain caveats. In two different IgE reporter mice, expression of membrane IgE is marked by green fluorescent protein (GFP) that is translated from a bicistronic IgE-IRES-GFP mRNA [16–18]. In these mice, GFP also reports germline, immature, and nonproductive transcripts. Therefore, a substantial fraction of GFP+ GC B cells actually express IgG1 and not IgE on the cell surface. One of these mouse strains also contains an insertion of a 52 amino acid region that is normally present in the extracellular part of human but not mouse IgE [18]. Another strain, the Verigem mouse [15], was constructed to express a membrane IgE-2A-Venus fusion protein that is cleaved into IgE and Venus, a brightly fluorescent protein. In this mouse strain, only mature transcripts are reported, but a 2–3-fold increase of membrane IgE and a reduction of secreted IgE has been noted.

On the molecular level, IgE-CSR was found to either occur directly from IgM to IgE or sequentially from IgM to IgG1 followed by a second switch reaction to IgE [19]. The relevance of the sequential switching pathway was questioned by experiments demonstrating that genetically modified mice that cannot switch to IgG1 show the same serum IgE levels after primary *N*. *brasiliensis* infection as compared to control mice, but the memory IgE response was not investigated [20]. Furthermore, sequential switching was reported to be important for generation of high affinity IgE antibodies under rather nonphysiological conditions by either repeated immunizations of BALB/c mice with the hapten antigen NP-KLH or after OVA-PEP1 immunization of T/B monoclonal mice where all B cells are specific for influenza hemagglutinin and all T cells are specific for chicken ovalbumin [14,21,22]. Sequentially switched B cells can be identified with a quantitative polymerase chain reaction (PCR) assay that detects remnants of the Sγ1 region in the recombined Sμ-Sε allele [14]. However, switch remnants can also be present in the nonproductively rearranged allele, and only a fraction of the recombined switch regions retains Sγ1 DNA [21]. This PCR assay cannot provide information about the total frequency and relatedness of the original IgG1 repertoire and the sequentially switched IgE repertoire. However, this information can be obtained, as we show here, by next generation sequencing (NGS) of RT-PCR products that cover the recombined variable, diversity, and joining (VDJ) regions and the 5’ part of the Cγ1 or Cε genes, respectively.

The question whether bona fide IgE^+^ memory B cells do exist or not continues to be controversially discussed [23]. Mice with deletion of the transmembrane and cytoplasmic tail of IgE (ΔM1M2 mice) show a poor memory IgE response indicating that bona fide IgE^+^ memory B cells exist and directly respond to antigen challenge [24]. However, the IgE response was also reduced 10-fold after primary infection of ΔM1M2 mice, suggesting that these mice have a general defect to mount IgE responses [24]. Others have shown that transfer of GFP^+^-sorted memory B cells from *N*. *brasiliensis*-infected IgE-GFP reporter mice into B cell-deficient hosts gave rise to serum IgE levels after *N*. *brasiliensis* infection [16]. However, in these reporter mice, GFP appears to be expressed also by some IgG1^+^ B cells, which may have contaminated the population of transferred B cells, and these mice express an engineered membrane IgE molecule that contains 52 amino acids of human IgE, which may alter the behavior of IgE^+^ B cells [25]. Studies with other IgE reporter mice or transfer of purified IgG^+^ memory B cells from T/B monoclonal mice indicated that the memory IgE response does not develop from bona fide IgE^+^ memory B cells but rather depends on an IgG1^+^ precursor population [14,15,17].

Using NGS analyses, we observed a striking overlap between the IgG1 and IgE repertoires in *N*. *brasiliensis*-infected or OVA/alum-immunized wild-type BALB/c mice. Competitive adoptive transfers further revealed that T cell-derived IL-4/IL-13 was required for the memory IgE response but not for expansion of memory B cells. IgG1^+^ B cells were required and sufficient to establish the memory IgE response after transfer in IgH allogeneic recipients. Interestingly, the memory IgE response was also impaired in mice where the extracellular parts of IgG1 had been replaced by IgE domains. Collectively, our results demonstrate that the memory IgE response is largely dependent on clonal expansion and affinity maturation of IgG1-expressing B cells that require a second IL-4/IL-13 signal from T cells to subsequently switch to IgE and differentiate into IgE-secreting plasma cells.

## Results

### Formation of IgE^+^ and IgG1^+^ B Cells and Plasma Cells Requires T Cell-Derived IL-4/IL-13

Mice with selective deletion of IL-4/IL-13 in T cells (4-13Tko mice) are unable to mount a serum IgE response and show impaired GC formation after helminth infection [9,26]. To further address how T cell-derived IL-4/IL-13 promotes development of IgE^+^ B cells and plasma cells (PCs) in vivo, we analyzed wild-type, IL-4/IL-13-deficient (4-13ko), and 4-13Tko mice after *N*. *brasiliensis* infection by flow cytometry.

**Fig 1:**
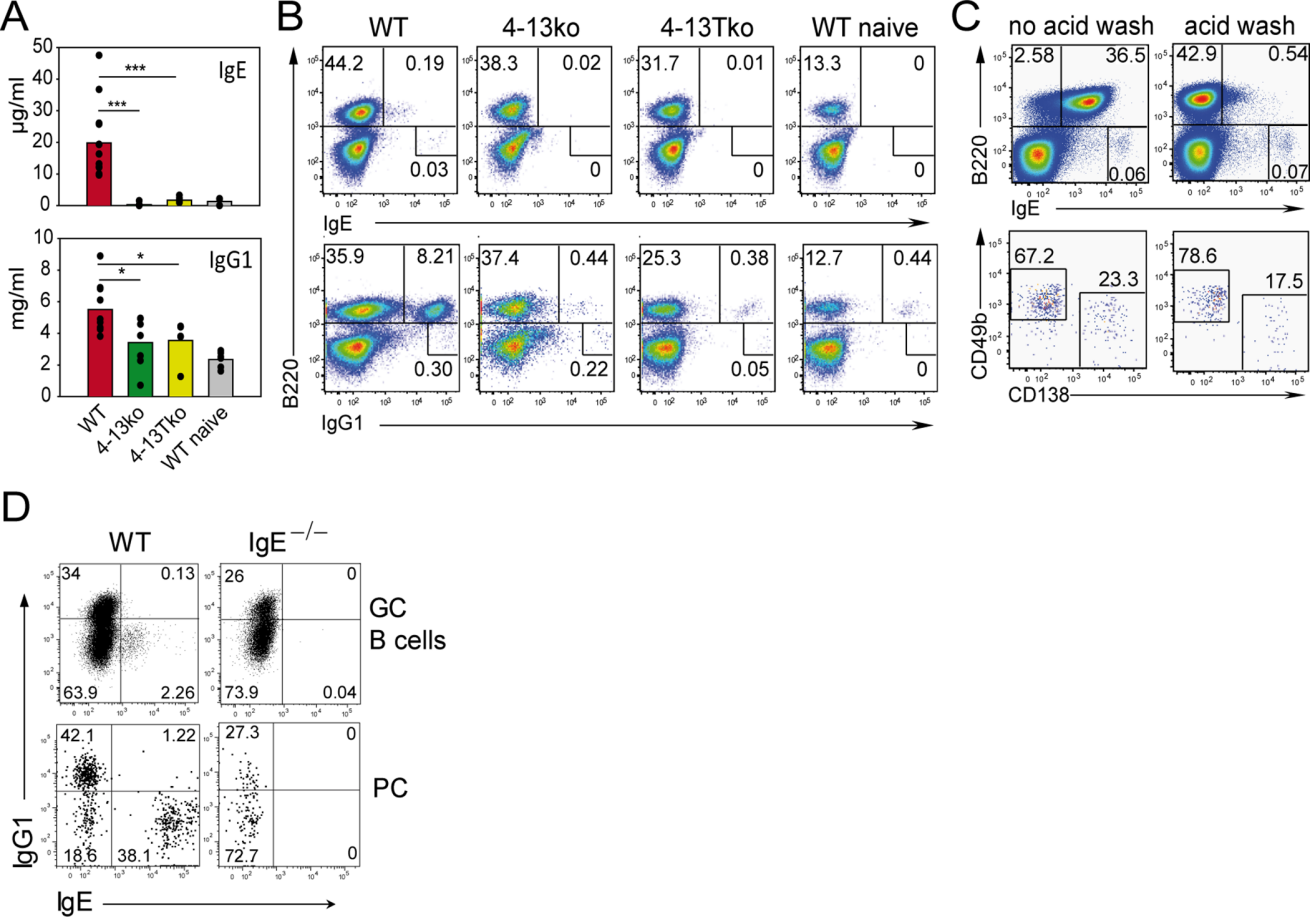
IL-4/IL-13 secreted by CD4+ T cells is necessary for the IgE and IgG1 response. Serum (A) and mesenteric LN (B and C) of WT, IL-4/IL-13 double deficient mice (4-13ko), or T cell-specific conditional IL-4/IL-13-deficient mice (4-13Tko) were collected from naïve mice or on day 12 after Nb infection. (A) Serum ELISA for total IgE and IgG1. Bars show the mean of individual mice (dots). (B) Cells were washed with acidic buffer to remove cytophilic IgE and permeabilized before staining. Dot plots are gated on live cells as indicated in S8 Fig and show the frequency of B cells (B220^+^) or B220^−^ cells expressing either IgE or IgG1. (C) Surface staining of B220 and IgE before (left plots) or after (right plots) acid wash in dot plots gated as indicated in S8 Fig. The upper dot plots demonstrate the reduction of cytophilic IgE after wash with acidic buffer on B220^+^ but not on B220^–^ cells. The lower plots are gated on B220^–^IgE^hi^ cells and show basophils (CD138^–^CD49b^+^) and plasma cells (CD138^+^CD49b^−^). (D) Intracellular staining for IgG1 and IgE after acid wash in GC B cells (B220^+^CD38^−^GL-7^+^) and plasma cells (PC, B220^lo^CD138^+^) from LN on day 10 after secondary Nb infection of BALB/c (WT) and IgE-deficient (IgE^−/−^) mice gated as indicated in S9 Fig. *p < 0.05, ***p < 0.001 by Student’s *t* test. Data are from at least four mice and at least two independent experiments.

We first treated cells isolated from the draining lymph nodes (LN) with an acidic wash buffer to remove cytophilic IgE bound to the low affinity IgE receptor FcεRII (CD23) and then stained for surface and intracellular IgE and IgG1. In our hands, this procedure is comparable to an alternative IgE staining protocol where extracellular IgE is first blocked by anti-IgE antibodies followed by intracellular staining for IgE [15] (S1 Fig). We observed that IgE^+^ B cells and PCs were missing in 4-13ko and 4-13Tko mice compared to wild-type (WT) controls, and IgG1^+^ B cells were reduced 20-fold (Fig 1A and 1B). It is important to note that the acidic wash buffer efficiently removed cytophilic CD23-bound IgE from B cells and PCs, as the vast majority of IgE^+^ GC B cells and PCs was restricted to the IgG1-negative population and was not found in IgE-deficient mice (Fig 1C and 1D). The acidic wash buffer did not affect IgE bound to the high affinity IgE receptor FcεRI, which is mainly expressed on basophils and mast cells (Fig 1C). We observed that the B220^−^IgE^+^ cells found in the LN of infected WT mice are composed mostly of basophils (80%; CD49b^+^CD138^−^) and by a smaller part of PCs (20%; CD49b^−^CD138^+^).

### IgE-Secreting PCs Develop Mainly from IgG1-Switched GC B Cells

**Fig 2:**
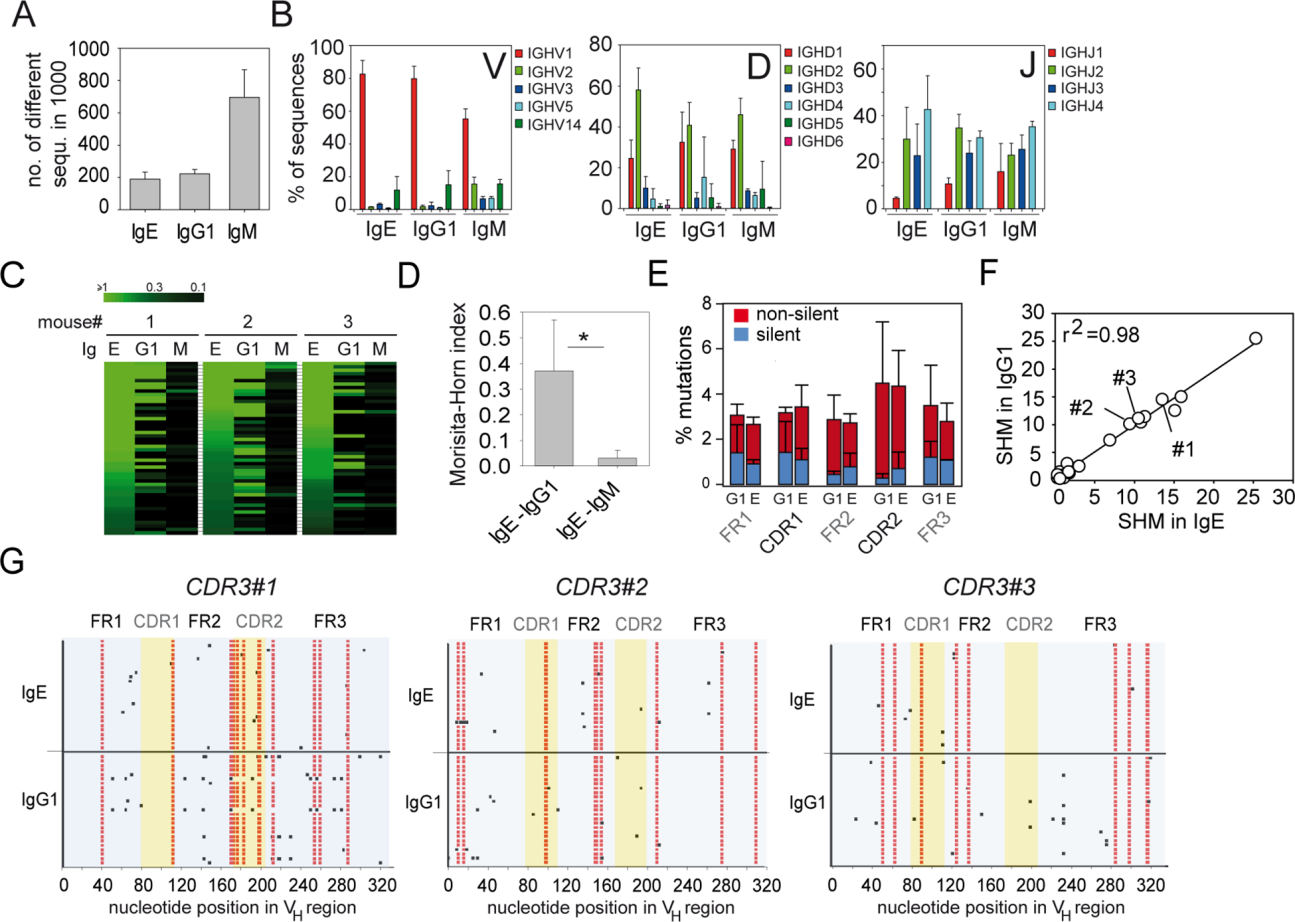
The IgE repertoire is closely related to the IgG1 repertoire. Analysis of IgE, IgG1, and IgM sequences from mediastinal LN of 3 mice 15 d after primary *N*. *brasiliensis* infection. (A) Repertoire diversity displayed as number of different CDR3 sequences among 1,000 randomly selected sequences. (B) Usage of different V_H_, D_H_, and J_H_ segments. (C) Heat maps show the overlap between the first 50 most frequent CDR3 sequences in the IgE repertoire with the same CDR3 sequences in the IgG1 and IgM repertoires from 3 mice. Each row indicates one unique CDR3 sequence ordered by decreasing frequency within all analyzed IgE sequences. The brightest green indicates CDR3 sequences with an abundance of ≥ 1%. (D) Morisita-Horn indices show the relatedness (0 = unrelated; 1 = identical) between 1,000 randomly selected CDR3 sequences of the IgE and IgG1 repertoires or IgE and IgM repertoires (mean + standard deviation [SD], *n* = 3). (E) Mean frequency + SD of silent (blue) and nonsilent (red) mutations in V_H_ segments of IgG1 and IgE. (F) Correlation between the average number of somatic hypermuations (SHMs) in 15 randomly selected V_H_ sequences from 25 pools of IgE and IgG1 sequences that share the same CDR3. r^2^ = linear regression analysis. (G) SHMs in 25 V_H_ segments of IgG1 and IgE from three different pools of sequences that share the same CDR3 (indicated in F). Mutations shared between IgE and IgG1 repertoires are shown in red. Additional mutations are shown in black. *p < 0.05 by Student’s *t* test.

Class switch recombination to IgE can occur directly from an IgM^+^ B cell or sequentially via an IgG1^+^ B cell intermediary [27]. We reasoned that sequential switching should be reflected by overlapping repertoires of IgE and IgG1 sequences. To address this experimentally, we analyzed thousands of VDJ regions from the heavy chains of IgE, IgG1, and IgM by NGS using a similar approach as we previously described for analysis of the intestinal IgA repertoire [28]. Reverse transcription PCRs (RT-PCRs) were performed with RNA samples from total mediastinal LN cells on day 15 after *N*. *brasiliensis* infection using a promiscuous 5’ primer that binds to VH1, 2, 3, 5, and 14 family sequences and thereby picks up most of the expressed VH genes [29] in combination with 3’ primers that bind in the Cε, Cγ1 and Cμ genes. The amplified VDJ sequences were then subjected to NGS analysis. We analyzed several thousand sequences per isotype and determined the richness as a measure of repertoire diversity. We observed a 3-fold higher richness in the IgM repertoire as compared to the IgE and IgG1 repertoires reflecting clonal expansion of isotype-switched B cells (Fig 2A). The usage of different V_H_, D_H_, and J_H_ segments was very similar among the three isotypes and the majority of sequences used in the V_H1_/J558 family [30] in combination with D_H1_ or D_H2_ families (Fig 2B). We further determined the relative abundance of individual CDR3 sequences within the total IgE, IgG1, and IgM sequences from each mouse. A striking overlap was found between CDR3 regions of IgE and IgG1 sequences within but not between individual mice (Fig 2C and 2D). In contrast, little overlap of CDR3 sequences existed between the IgE and IgM repertoires (Fig 2C and 2D). We obtained similar results with samples from mediastinal LN of OVA/alum-immunized mice (S2 Fig) and samples from mesenteric LN of five independently *N*. *brasiliensis*-infected mice (S3 Fig). In contrast to the prominent IgE-IgG1 overlap, we found only little overlap between IgA and IgG1 or IgM repertoires or between IgE and IgA repertoires (S3D and S3E Fig).

**Fig 3:**
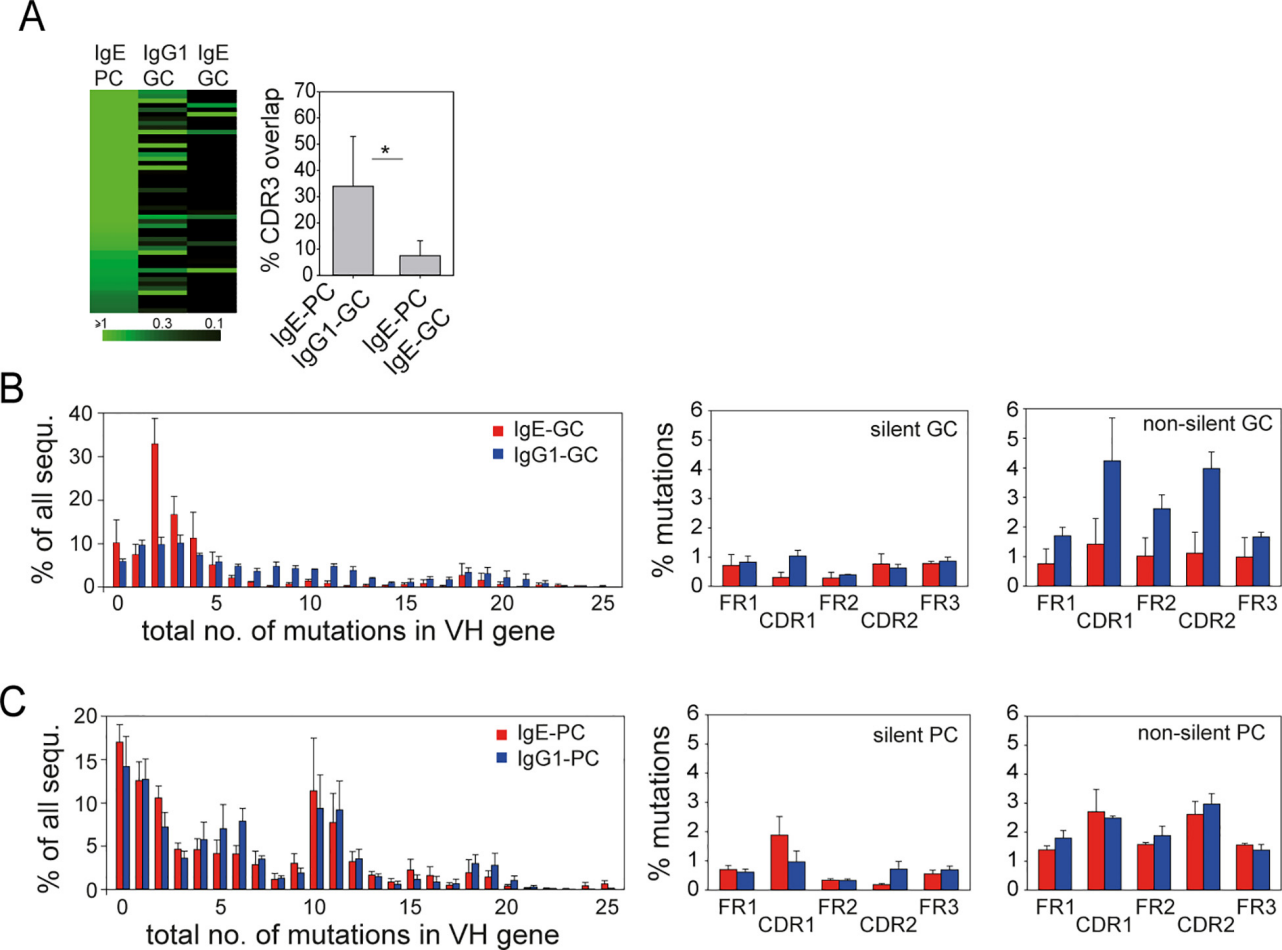
The repertoire of IgE^+^ PCs is more closely related to IgG1^+^ GC B cells than IgE^+^ GC B cells. (A) Overlap between the 50 most frequent CDR3 sequences in IgE^+^ PCs with the same CDR3 sequences in the IgG1^+^ or IgE^+^ GC B cell repertoire. Bars show the mean frequency + SD of CDR3 sequences in IgE^+^ PCs for which corresponding CDR3 sequences were detected in the IgE^+^ or IgG1^+^ GC B cell repertoires. *p < 0.05 by Student’s *t* test. (B and C) Frequency of SHMs and distribution of silent and nonsilent mutations in VH sequences of sorted IgE-GC (red) and IgG1-GC (blue) B cells (B) or IgE-PC (red) and IgG1-PC (blue) (C). Bars show the mean + standard error of the mean (SEM) of samples from three individual mice.

Comparison of somatic hypermutations (SHMs) between the IgE and IgG1 repertoire revealed a similar average number of SHMs in different parts of the V_H_ regions of IgE and IgG1 sequences (Fig 2E and S2 Fig). The average number of SHMs in V_H_ regions of corresponding CDR3 pools of the IgE and IgG1 repertoire were basically identical (Fig 2F). Alignments of the patterns of SHMs from three different corresponding CDR3 pools of IgE and IgG1 sequences showed that the core pattern of SHMs was very similar for each CDR3 pool. However, sequences with additional mutations could be observed for both isotypes (Fig 2G). To figure out whether the repertoire of IgE^+^ PCs was closer related to IgE^+^ or IgG1^+^ GCs we sorted these populations from LN of infected mice and determined whether the CDR3 sequences that constituted the first 50 most abundant CDR3 pools in the IgE^+^ PC population also show up in the IgE^+^ GC or IgG1^+^ GC population. This analysis revealed that the IgE^+^ PC repertoire is more closely related to the IgG1^+^ GC as compared to the IgE^+^ GC repertoire (Fig 3A). IgE^+^ GC B cells contained relatively few nonsilent SHMs, indicating that they did not undergo affinity maturation (Fig 3B and 3C).

Taken together, these findings clearly demonstrate that the sequential pathway for IgE-CSR dominates the in vivo IgE response and points to an important role of the GC as the site where affinity maturation probably occurs at the level of IgG1-expressing B cells that have the capacity to further switch and differentiate to IgE-producing PCs. This raises the question whether memory IgE responses are driven by IgE^+^ memory B cells or rather depend on memory IgG1^+^ B cells that switch to IgE after secondary antigen encounter.

### Increased Number of PCs, Lack of a GC Response, and Dependence on CD4^+^ T Cell Help Characterizes the IgE Memory Response

**Fig 4:**
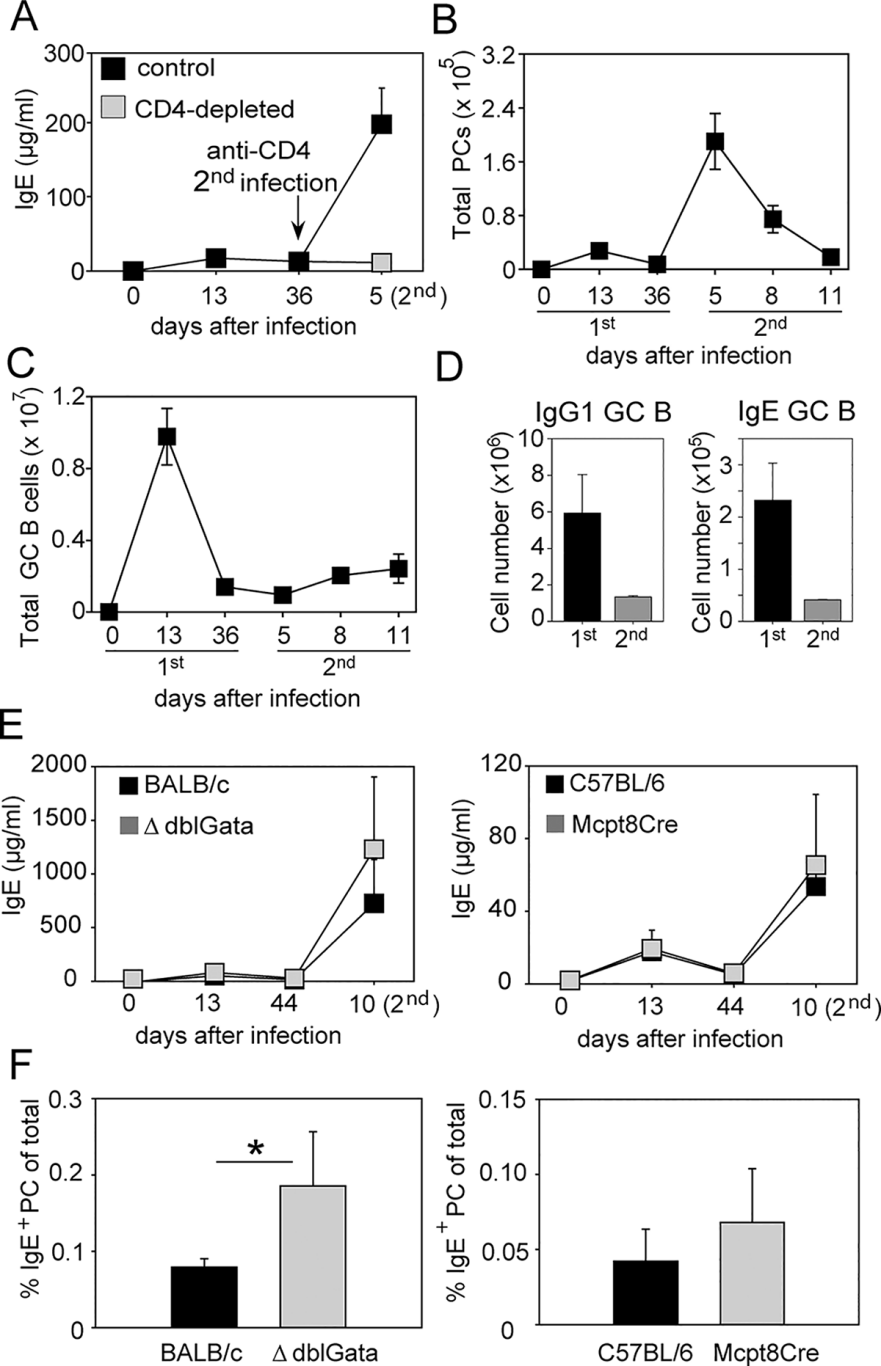
IgE memory response to *N*. *brasiliensis* is characterized by increased numbers of PCs, lack of a GC response, and dependence on CD4^+^ T cell help. (A) Serum IgE in BALB/c mice after first and secondary *N*. *brasiliensis* infection (black squares). One group of mice was injected with CD4-depleting antibody (400 μg clone GK1.5; BioXCell) for three consecutive days starting three days before the second infection (grey square). The total number of PCs (B220^–^CD138^+^FSC^hi^SSC^hi^; shown in (B)) and GC B cells (B220^+^CD38^lo^GL-7^hi^; shown in (C)) were determined in pooled mesenteric and mediastinal LN samples along the course of the primary and secondary *N*. *brasiliensis* infection. (D) Number of IgG1^+^ and IgE^+^ GC B cells at day 13 after primary, and day 11 after secondary, *N*. *brasiliensis* infection. (E) Serum IgE was measured after primary and secondary *N*. *brasiliensis* infection of eosinophil-deficient ΔdblGata mice (grey squares, left graph) or basophil-deficient Mcpt8Cre mice (grey squares, right graph) and their corresponding control mice (black squares). (F) Frequency of IgE^+^ PCs (B220^–^CD138^+^IgE^+^) was determined at day 10 after secondary *N*. *brasiliensis* infection in pooled mediastinal and mesenteric LN samples of indicated mice. Data show the mean ± SEM from at least two independent experiments with 3–5 mice per group. The full gating strategy for GC B cells and PCs is shown in S9 Fig. *p < 0.05 by Student’s *t* test.

Consistent with previous reports, we observed that the serum IgE concentration reaches about 10-fold higher levels after secondary as compared to primary infection with *N*. *brasiliensis*, and this effect was dependent on the presence of CD4^+^ T cells [10,31] (Fig 4A). We further found that this increase in serum IgE was accompanied by a 6-fold increase of PCs (B220^−^CD138^+^FSC^hi^SSC^hi^) when compared to the primary response (Fig 4B). Interestingly, no expansion of GC B cells (B220^+^CD38^lo^GL-7^hi^) was observed after secondary *N*. *brasiliensis* infection, indicating that the new antibody-secreting cells originate mostly from pre-established memory B cells (Fig 4C and 4D). Eosinophils and basophils were reported to contribute to the memory response by promoting plasma cell survival in the bone marrow and spleen, respectively [32,33]. However, we observed unimpaired memory IgE responses in eosinophil-deficient ΔdblGata [34] and basophil-deficient Mcpt8Cre mice [35] (Fig 4E and 4F).

**Fig 5:**
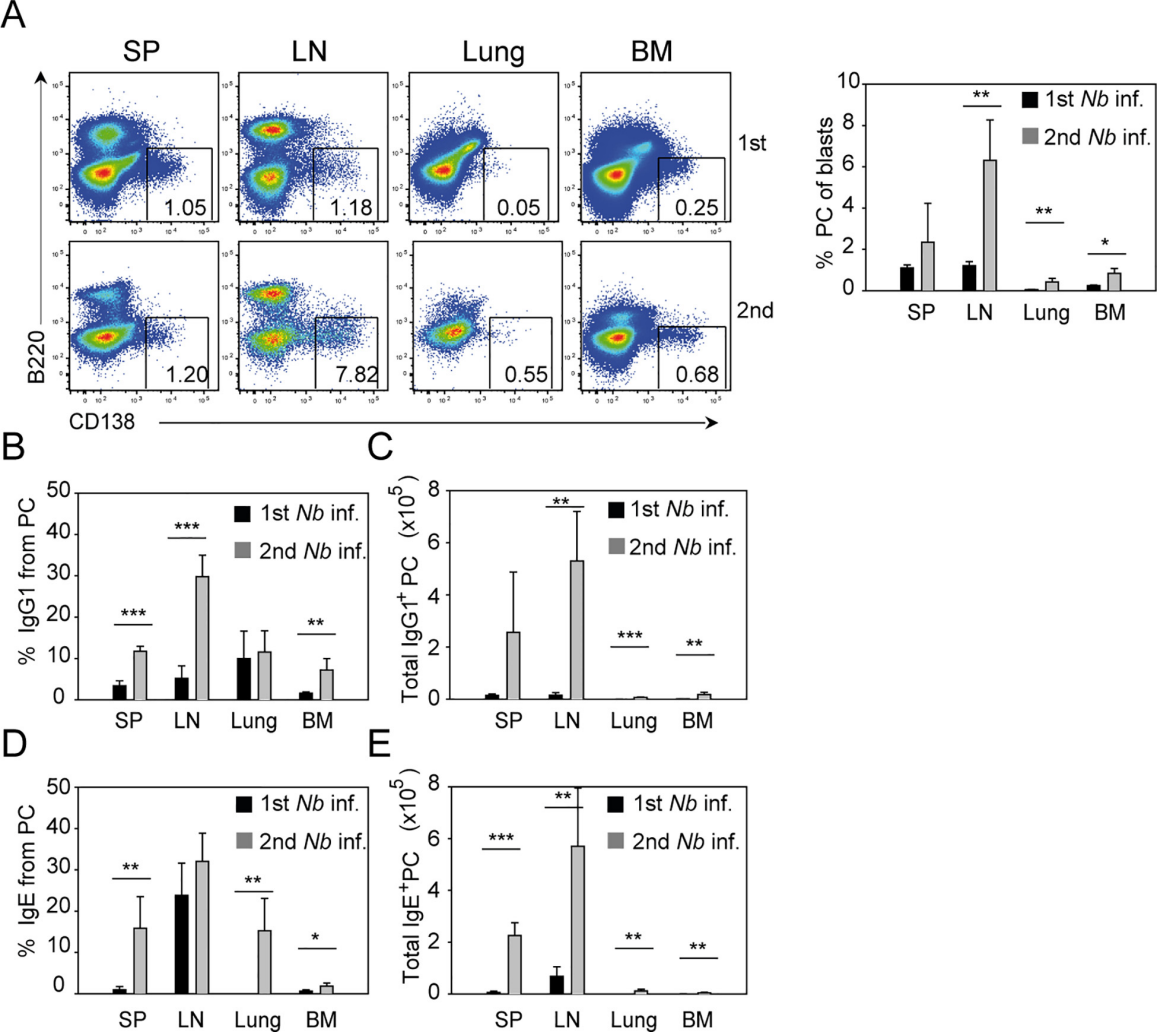
IgE^+^ and IgG1^+^ PCs increase in numbers in different organs after secondary *N*. *brasiliensis* infection. Spleen (SP), pooled mediastinal, and mesenteric LN, lung, and bone marrow (BM) of BALB/c mice were collected 13 d or 10 d after primary and secondary *N*. *brasiliensis* infection, respectively. (A) Representative plots show the percentage of PCs (B220^–^CD138^+^) from blasts (FSC^hi^SSC^hi^) after primary (upper plots) and secondary (lower plots) *N*. *brasiliensis* infection gated as indicated in S10 Fig. Bar graph shows the average percentage of PCs in indicated tissues. (B–E) Bar graphs show the mean percentage of IgG1^+^ PCs (B), total IgG1^+^ PCs (C), percentage of IgE^+^ PC (D) and total IgE^+^ PC (E) in indicated tissues using the PC gate shown in Fig 5A and intracellular staining for IgG1 and IgE as shown in S11 Fig. Data show the mean + SEM from three independent experiments and at least six mice. *p < 0.05, **p < 0.005, ***p < 0.001 by Student’s *t* test.

Next, we investigated the distribution of IgE- and IgG1-secreting PCs in different tissues after the first and second *N*. *brasiliensis* infection. At day 13 after primary infection, the peak of the humoral response, PCs could be found mainly in the draining LN and spleen of infected mice (Fig 5A). After secondary infection, the total number of PCs mainly increased in the LN and to a lesser extent in lung, spleen, and bone marrow (Fig 5A). IgE^+^ and IgG1^+^ PCs were found in the spleen and LN of primary infected mice, and in the memory response they became much more abundant in these organs, whereas IgE^+^ and IgG1^+^ PCs in the bone marrow and lung remained relatively scarce, although their numbers increased during the secondary response (Fig 5B–5E).

### Comparative Analysis of IgE Repertoires in Different Tissues

**Fig 6:**
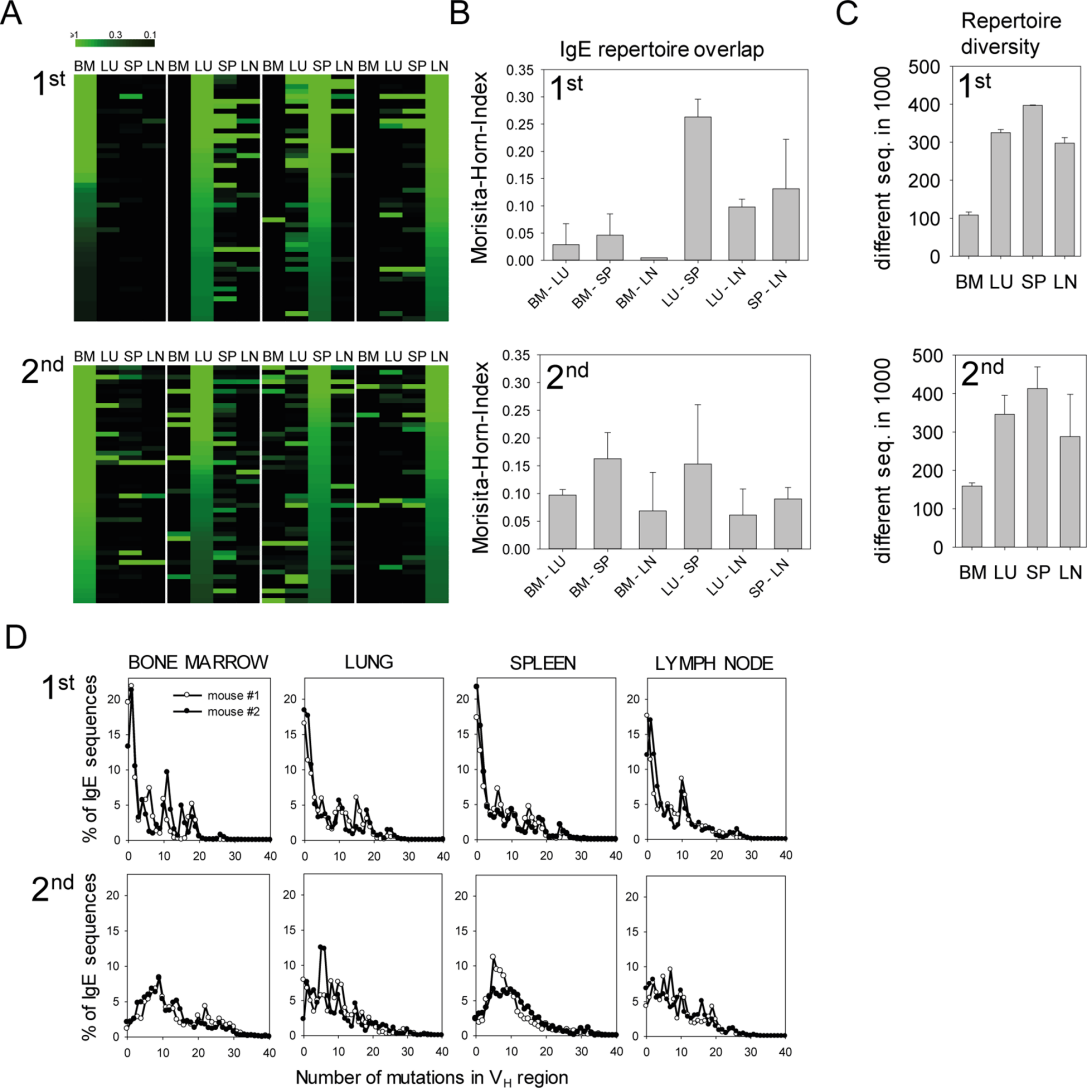
Overlapping IgE repertoires in different organs after primary and secondary *N*. *brasiliensis* infection. (A) Heat maps for the first 50 most frequent CDR3 sequences in the IgE repertoires from bone marrow (BM), lung (LU), spleen (SP), and lymph nodes (LN) of one exemplary *N*. *brasiliensis*-infected mouse after primary (1st) and secondary infection (2nd). Each row indicates one unique CDR3 sequence ordered by decreasing frequency in the IgE pools of BM, LU, SP, and LN (left to right). The brightest green indicates an abundance of ≥1% of a particular CDR3 sequence. (B) Morisita-Horn indices show the relatedness between 1,000 randomly chosen CDR3 sequences of IgE in the indicated organs after primary and secondary infection. (C) Number of different CDR3 in 1,000 randomly chosen IgE sequences from indicated organs after primary and secondary infection. (D) Frequencies of SHMs in V_H_ regions of IgE in indicated organs after primary and secondary infection. Data in (B) and (C) show the mean + SEM from two mice per experiment.

We further analyzed the overlap of the IgE repertoires in bone marrow, lung, spleen, and LN after primary and secondary infection by NGS analysis to determine the clonal dissemination of IgE^+^ PCs in these tissues. During primary infection, the IgE repertoires in lung, LN, and spleen showed pronounced overlaps, while the IgE repertoire in the bone marrow was rather unique. However, an increased overlap between the IgE repertoire in the bone marrow and the other organs was observed after secondary infection (Fig 6A and 6B). The IgE repertoires had a high diversity with 300–400 different sequences among 1,000 analyzed sequences in lung, spleen, and LN during primary or secondary infection, whereas the diversity was 2–3-fold lower in the bone marrow (Fig 6C). Similar to the primary infection, the majority of sequences used the V_H1_/J558 family in combination with D_H1_ or D_H2_ segments (S4 and S5 Fig). By analyzing the number of SHMs in the V_H_ region of productive sequences, we observed that during primary infection about 50% of all IgE sequences in all organs contained 0–3 SHMs. During secondary infection, the repertoires were dominated by sequences with 3–20 SHMs reflecting further selection and affinity maturation (Fig 6D).

### Functional IgE Memory Is Mainly Localized in Spleen and LN

**Fig 7:**
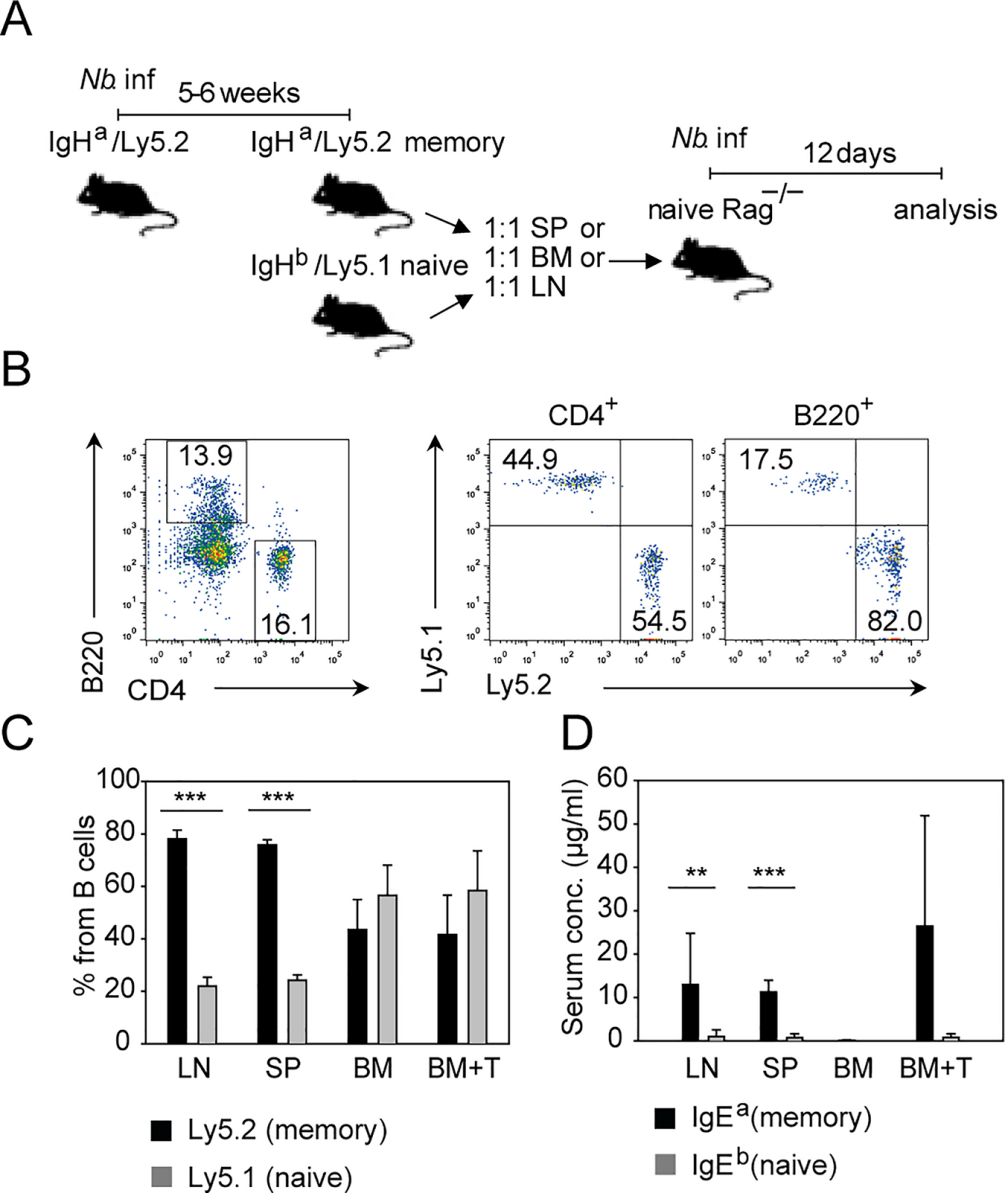
IgE memory resides mainly in spleen and LN. (A) Outline of transfer experiment referring to data in B–D. IgH^a^/Ly5.2 mice were infected with *N*. *brasiliensis* 5–6 wk before cell transfer to establish memory mice. Cell suspension from SP, BM, or LN from memory IgH^a^/Ly5.2 and naïve IgH^b^/Ly5.1 mice were mixed at a 1:1 ratio of B cells from each mouse and transferred into Rag1^–/–^mice. Mesenteric LN and serum were analyzed 12 d after *N*. *brasiliensis* infection of Rag1^−/−^ recipient mice. (B) Representative plots showing transferred CD4^+^ T cells and B220^+^ B cells (left) and percentage of naïve (Ly5.1^+^) and memory (Ly5.2^+^) CD4^+^ T cells (middle plot) or B220^+^ B cells (right plot) gated as indicated in S8 Fig. (C) Bar graph shows the percentage of B cells from naïve or memory donor cells from LN, SP, or BM in the mesenteric LN of infected Rag1^−/−^ recipient mice. BM+T correspond to the transfer of 1:1 B cells from memory and naïve mice plus 1 part purified CD4^+^ T cells from naïve mice. (D) Bar graph shows IgE produced by B cells from memory mice (detected as IgE^a^) or B cells from naive mice (detected as IgE^b^) in the serum of infected Rag1^–/–^recipient mice. Data show the mean + SEM from two independent experiments and 5–6 mice per group (LN, SP, BM) or 2 mice per group (BM+T). **p < 0.005 ***p < 0.001 by Student’s *t* test.

Since IgE^+^ PCs with overlapping repertoires could be found in spleen, mesenteric LN, and bone marrow after secondary *N*. *brasiliensis* infection, we sought to evaluate how well memory IgE precursor cells from these organs perform in a competitive transfer experiment with naïve cells. For this purpose, we used congenic Ly5 mice expressing different immunoglobulin heavy chain allotypes. First, we infected Ly5.2 mice that carried the Ig heavy chain of the “a” allotype (Ly5.2/IgH^a^ mice) with *N*. *brasiliensis*. Five to six weeks later, we isolated total lymphocytes from LN, spleen, and bone marrow of these *N*. *brasiliensis* memory mice and the corresponding cell populations from naïve Ly5.1 mice that were of the Ig heavy chain “b” allotype (Ly5.1/IgH^b^ mice). The samples from naïve and memory mice were adjusted to equal numbers of B cells, mixed, and transferred to Rag1^–/–^mice, which were infected with *N*. *brasiliensis* 1 d later (Fig 7A). For the bone marrow samples, we reasoned that the memory response might be poor because of the relatively low number of CD4^+^ T cells in these organs. Therefore, we also included one group of mice that received purified CD4^+^ T cells from the spleen of naïve WT mice in addition to bone marrow cells from naïve and *N*. *brasiliensis* memory mice. The B cells from LN and spleen of memory mice expanded 4–5 times better as compared to cotransferred B cells from naïve mice, while both populations expanded with the same efficiency when they were derived from the bone marrow, even in the group that received additional purified CD4^+^ T cells (Fig 7B and 7C). The dominance of B cells derived from spleen or LN of memory mice was confirmed in the reverse experiment with memory Ly5.1/IgH^b^ and naïve Ly5.2/IgH^a^ donors (S6 Fig). Furthermore, B cells derived from memory mice mainly outcompeted B cells derived from naïve mice in the CD38^+^IgD^−^ B cell population (largely reflecting memory B cells) as compared to the CD38^+^IgD^+^ population (largely reflecting naïve B cells) (S6 Fig). Most IgE antibodies detected in the serum were secreted by cells that originated from LN or spleen of memory mice (Fig 7D). In contrast, a memory IgE response from bone marrow samples was only observed when purified CD4^+^ T cells had been cotransferred (Fig 7D). This indicates that IgE memory precursor cells are present in the bone marrow, but there is not enough help provided by the few CD4^+^ T cells in this organ. Although addition of exogenous T cells did not improve the expansion of B cells derived from bone marrow of memory mice, it induced the production of IgE from memory-mice-derived B cells, suggesting that memory B cells require T cell signals to further differentiate into IgE-producing plasma cells.

### T Cell-Derived IL-4/IL-13 Is Required for the Memory IgE Response but Not for Expansion of Memory B Cells

**Fig 8:**
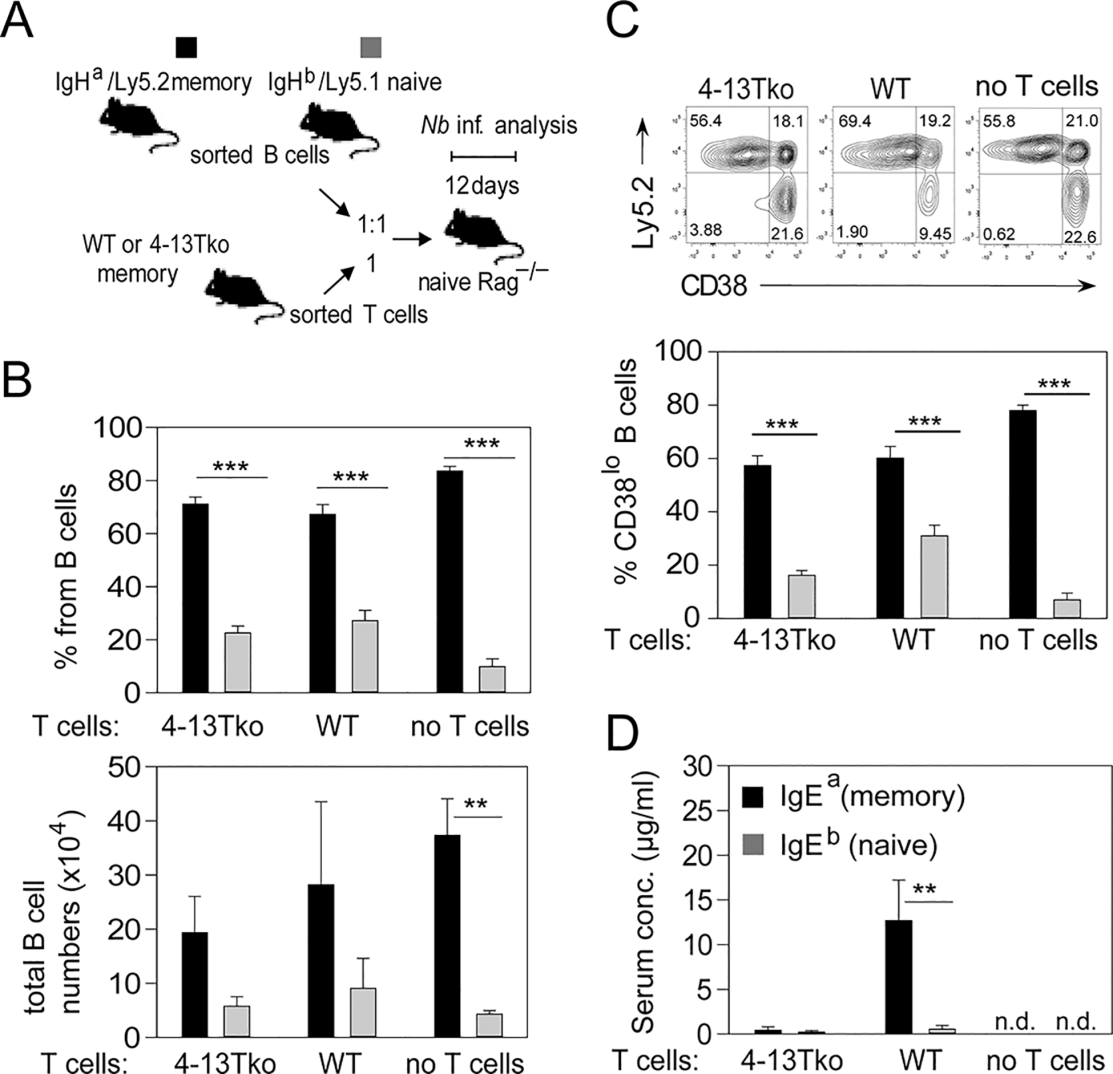
*N*. *brasiliensis*-elicited memory B cells depend on IL-4/IL-13 secretion from CD4^+^ T cells for IgE secretion but not for expansion. (A) Outline of transfer experiment referring to data in B, C, and D. B cells from memory IgH^a^/Ly5.2 and naïve IgH^b^/Ly5.1 mice were sorted and transferred into Rag1^–/–^mice without or with sorted WT or 4-13Tko memory T cells. Mesenteric LN and serum were analyzed 12 d after *N*. *brasiliensis* infection of Rag1^–/–^recipient mice. (B) Percentage and total numbers of B220^+^ cells originated from the memory (Ly5.2, black bars) or naïve (Ly5.1, grey bars) mice in Rag1^–/–^mice that also received either 4-13Tko or WT memory T cells, or no T cells gated as indicated in S12A Fig. (C) Expression of CD38 on B220^+^ cells from memory mice (Ly5.2^+^, black bars) or naïve mice (Ly5.2^−^, grey bars) without or with cotransfers of T cells from 4-13Tko or WT memory mice gated as indicated in S12B Fig. (D) Bar graph shows IgE secreted from memory (detected as IgE^a^) or naive cells (detected as IgE^b^) in the serum of Rag1^–/–^mice that received either 4-13Tko or WT memory T cells. Data show the mean + SEM from 2–3 independent experiments and 4–8 mice per group. **p < 0.005 ***p < 0.001 by Student’s *t* test. n. d. = not detectable.

To further elucidate whether T cell-derived IL-4 was required for the IgE memory response, we transferred 2 x 10^5^ purified CD4^+^ T cells from WT or 4-13Tko *N*. *brasiliensis* memory mice together with 2 x 10^5^ purified and equally mixed B cells from memory IgH^a^ and naïve IgH^b^ mice into Rag1^−/−^ recipients (Fig 8A). As additional control, one group of mice only received mixed B cells but not T cells. B cells derived from memory mice expanded four times better than B cells from naïve mice independently of cotransferred CD4^+^ T cells (Fig 8B). In addition, B cells from memory mice appeared more activated based on CD38 down-regulation as compared to B cells from naïve mice independently of T cells (Fig 8C). However, IgE was secreted in the serum only by cells originating from the memory mice and only if IL-4/IL-13 competent T cells were cotransferred along with the B cells (Fig 8D). This indicates that T cell-derived IL-4/IL-13 promotes the secondary switch and differentiation of IgG1^+^ memory B cells into IgE-secreting PCs.

### The Extracellular Part of IgG1 Is Required for the Memory IgE Response

**Fig 9:**
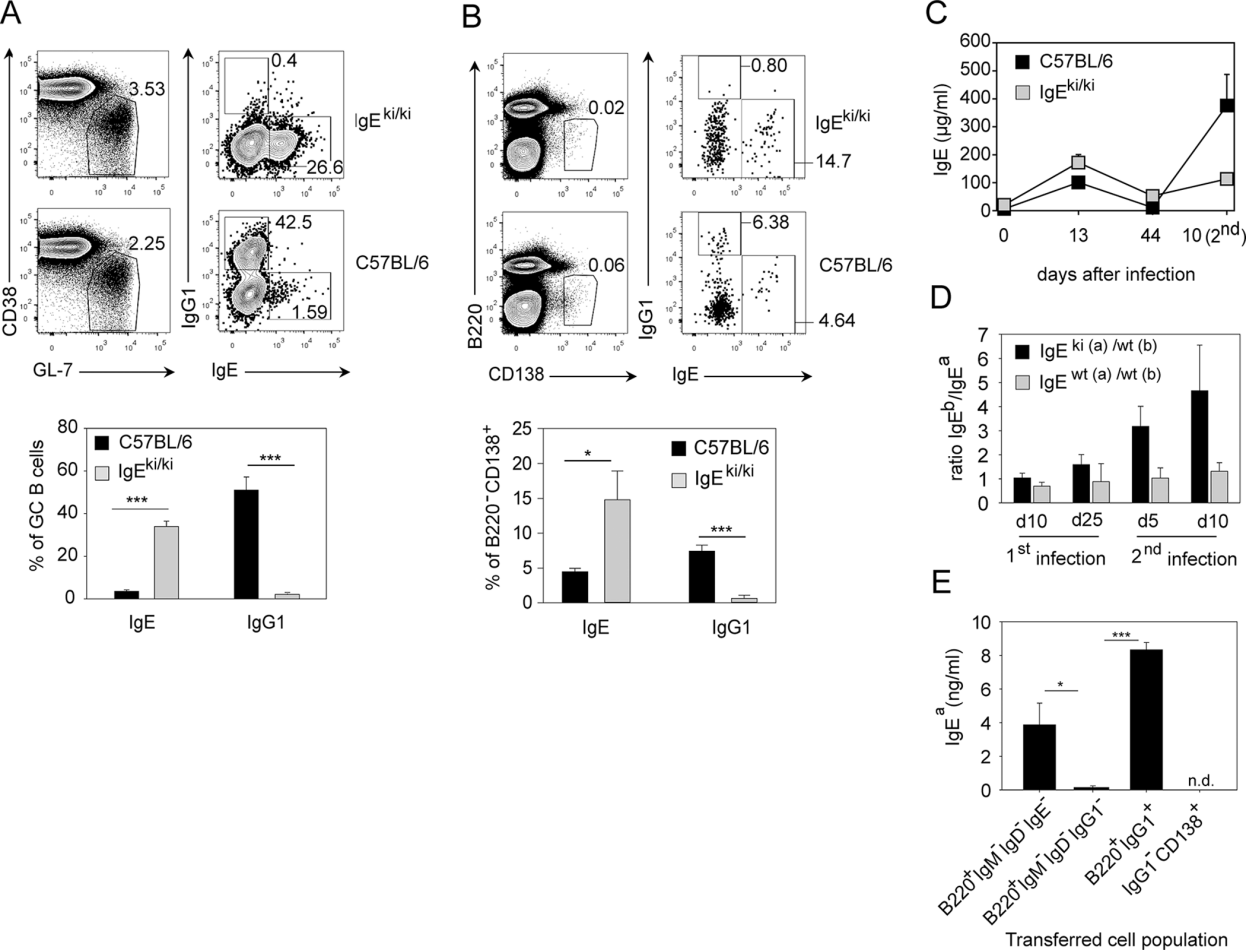
IgG1^+^ precursors are required for constitution of the memory IgE response. (A) GC B cells (B220^+^CD38^lo^GL-7^+^; left plots) and intracellular IgE^+^ or IgG1^+^ expression on gated GC B cells (right plots) in mesenteric LN samples from day 14 *N*. *brasiliensis*-infected IgE^ki/ki^ and wild-type C57BL/6 mice. (B) PCs (B220^−^CD138^+^; left plots) were gated from blasts (FSC^hi^SSC^hi^), and percentage of intracellular IgE^+^ and IgG1^+^ PCs is shown in the right plots. (C) Serum IgE in IgE^ki/ki^ and C57BL/6 mice after first and secondary *N*. *brasiliensis* infection. (D) Ratio of IgE^b^ to IgE^a^ in IgE^ki(a)/wt(b)^ or IgE^wt(a)/wt(b)^ mice after first and secondary *N*. *brasiliensis* infection. (E) 1.5 x 10^5^ sorted B220^+^IgM^−^IgD^−^IgE^−^, B220^+^IgM^−^IgD^−^IgG1^−^, B220^+^IgG1^+^ B cells or IgG1^−^CD138^+^ PCs gated as indicated in S7 Fig from IgH^a^ mice on d13 after secondary *N*. *brasiliensis* infection were transferred into nonirradiated IgH^b^ mice. Recipient mice were infected with *N*. *brasiliensis* and IgE^a^ was determined in the serum on day 12 after infection. Data show the mean + SEM from four independent experiments and at least seven mice (A and B), two experiments with eight mice total per group (C), two experiment with 2–7 mice (E). *p < 0.05, ***p < 0.001 by Student’s *t* test. n. d. = not detectable.

We performed further experiments to clarify whether the memory IgE response is dependent on an IgG1^+^ memory B cell. Here, we used the recently described IgE knock-in mouse (IgE^ki/ki^) in which the extracellular part of the IgG1 heavy chain had been replaced by the extracellular part of the IgE heavy chain [36]. When mesenteric LN were analyzed on day 14 after *N*. *brasiliensis* infection, wild-type mice had about 43% IgG1^+^ and 1.6% IgE^+^ GC B cells, while IgE^ki/ki^ mice had no IgG1^+^ and about 27% IgE^+^ GC B cells (Fig 9A). However, this 20-fold increased population of IgE^+^ GC B cells in IgE^ki/ki^ mice did not result in the same increase of IgE^+^ PCs (Fig 9B), and the serum IgE levels were only about 2-fold higher in IgE^ki/ki^ mice as compared to control mice at the peak of the primary response to *N*. *brasiliensis* (Fig 9C). Interestingly, serum IgE levels during the recall response in IgE^ki/ki^ mice were comparable to the IgE levels after primary infection and much lower compared to IgE levels in control mice (Fig 9C). We further analyzed the IgE response in F1 mice generated by crossing IgE^ki/ki^ mice (IgH^a^) or normal IgH^a^ mice to C57BL/6 mice (IgH^b^). Due to allelic exclusion, about 50% of B cells in these mice express the IgH^a^ allele while the other 50% express the IgH^b^ allele. We found that IgE^b^ dominated the memory IgE response to *N*. *brasiliensis* in IgE^ki(a)/wt(b)^ mice illustrating the competitive advantage of the wild-type allele encoding the extracellular domains of IgG1 (Fig 9D). Next, we sorted different B cell subsets and PCs from *N*. *brasiliensis*-infected IgH^a^ memory mice and transferred them separately into naïve nonirradiated IgH^b^ recipient mice. After *N*. *brasiliensis* infection of recipient mice, we observed a prominent IgE response in recipients of B220^+^IgG1^+^ and B220^+^IgM^−^IgD^−^IgE^−^ cells but not in recipients of B220^+^IgM^−^IgD^−^IgG1^−^ or IgG1^−^ PCs (Fig 9E and S7 Fig).

Taken together, this set of experiments indicates that the memory IgE response to *N*. *brasiliensis* unfolds from IgG1^+^ memory B cells and requires the extracellular part of IgG1.

## Discussion

A detailed understanding of the mechanisms that regulate memory IgE responses is critical to develop efficient therapeutic strategies against chronic allergic inflammation. Here, we used mice with a normal T and B cell repertoire and without modification of the IgE locus in combination with the well-established *N*. *brasiliensis* infection model or OVA/alum immunization to uncover important new insights of the primary and memory IgE response.

It has been shown that B cells primed with a high concentration of KLH/alum differentiate mainly into IgG1^+^ B cells and transfer of 10^7^ total B cells from KLH/alum immunized mice into IL-4-deficient recipients results in a poor IgE memory response [37]. We extend these findings by showing that IL-4/IL-13 from T cells played a critical role for the enhanced IgE response but not for expansion of memory B cells during secondary *N*. *brasiliensis* infection, suggesting that memory B cells require T cell-derived IL-4/IL-13 to further differentiate into IgE-producing PCs, but not for proliferation or survival.

Interestingly, the number of GC B cells did not increase during secondary infection, while PCs showed massive expansion. Neither eosinophils nor basophils were required for secondary PC expansion or for the memory IgE response, although both cell populations are sources of IL-4 and IL-13 and despite previous reports that described critical roles of eosinophils and basophils for PC survival in bone marrow and spleen, respectively [32,33]. The role of GCs for the IgE response is not well understood. A previous study reported that germline and postswitch IgE transcripts could be detected in GC B cells but most IgE^+^ cells were found outside the GC and these cells displayed a PC phenotype [14]. Principally, the IgE response can unfold in the absence of GCs as increased IgE levels can be observed upon immunization of mice that make a poor GC response like Bcl6-deficient mice [38]. Furthermore, spontaneously increased serum IgE levels are detected in MHC-II-deficient and T-lymphopenic mice [39] or in Omenn syndrome patients with reduced Rag1 or Rag2 activity [40]. These “natural” IgE antibodies have almost no SHMs and do not require GCs for their generation [39]. However, GCs probably play an important role for T cell-dependent IgE responses to allergens and helminths, especially for high-affinity IgE antibodies that are most likely generated by sequential CSR from IgG1^+^ B cells [14,21]. The decision to undergo direct or sequential switching might be regulated in part by the amount of antigen [41]. IgE^+^ GC B cells from CεGFP reporter mice showed lower B cell receptor (BCR) expression levels, a poor BCR signaling response, decreased expression of costimulatory molecules, and enhanced apoptosis suggesting that IgE^+^ GC B cells are prone to die rather than giving rise to memory B cells and PCs [17]. However, no information was given in this study regarding IgE BCR expression levels in CεGFP versus WT mice, which would be an important piece of information to exclude a possible negative effect of the reporter construct.

We further demonstrate that a prominent IgE^+^ GC B cell population is present in IgE^ki/ki^ mice indicating that surface IgE^+^ B cells can participate in the GC reaction and are not immediately prone to die. However, we also observed that IgE^+^ GC B cells contain fewer SHMs as compared to IgG1^+^ GC B cells indicating that affinity maturation is impaired in IgE^+^ GC B cells. Interestingly, IgE^ki/ki^ mice failed to mount a normal IgE memory response suggesting that the extracellular part of the chimeric IgE-IgG1 receptor does not allow the formation of memory B cells. One possible explanation for this finding would be that IgE^+^ B cells are actively removed or silenced in vivo, although the mechanisms behind such a scenario remain unclear. An alternative explanation would be that antigen-independent binding of the extracellular part of IgG1 to a yet-to-be-identified target structure induces a prosurvival signal in memory IgG1^+^ B cells, which then give rise to the memory IgE response. Achatz et al. demonstrated that the memory IgE response is blunted when B cells express transmembrane IgE with a truncated cytoplasmic tail favoring the concept that the memory IgE response develops directly from IgE^+^ B cells [24]. However, the primary IgE response is also affected in these mice, and this finding is not necessarily in conflict with the observation that the memory IgE response develops from IgG1^+^ precursors. For instance, it could well be that IgE-secreting PCs develop first from IgG1^+^ memory B cells and the cytoplasmic tail of IgE is then required for survival of IgE^+^ PCs since transmembrane IgE is clearly expressed on these cells [15].

We show here, to our knowledge, for the first time an extensive NGS analysis of the IgE and IgG1 repertoires in normal BALB/c mice after helminth infection or during allergic inflammation. We found largely overlapping IgE and IgG1 repertoires based on the comparison of CDR3 regions from several thousand IgE and IgG1 sequences. The analysis of SHMs in selected pools of IgE and IgG1 sequences with the same CDR3 region revealed that the majority of SHMs was identical in both isotypes. Furthermore, we found that the Ig repertoire of IgE-producing PCs was more closely related to IgG1^+^ GC B cells as compared to IgE^+^ GC B cells. Early IgE^+^ PCs show very few SHMs and are often generated in extrafollicular foci, while PCs with SHMs are thought to be derived from GC B cells that arise later during an immune response [42]. The large number of SHMs in IgE^+^ PCs of *N*. *brasiliensis-*infected mice indicates that the repertoire of these PCs was generated during the GC response. Taken together, this set of experiments provides strong evidence that the majority of the IgE response to primary *N*. *brasiliensis* infection in mice with a normal polyclonal B and T cell repertoire is constituted by PCs originating from sequentially switched B cells.

The IgE repertoires between lung, spleen, and LN were very similar after primary and secondary infection, while an IgE repertoire overlap between bone marrow and the peripheral organs could only be observed after secondary infection. We unexpectedly observed a high IgE repertoire diversity with 300–400 different sequences among 1,000 analyzed sequences in lung, spleen, and LN after primary and secondary infection. This shows that many different clones of IgE-expressing cells disseminate into different tissues and the diversity was maintained after secondary infection, excluding the possibility that only few memory B cells participate in the memory response. The average number of SHMs in IgE increased after secondary infection and was remarkably constant in all four tissues. The pool of IgE memory precursor cells (most likely a population of IgG1^+^ memory B cells) is probably established after the first infection and does not require a second GC phase, since we observed no GC response upon secondary infection.

It presently remains unclear whether the mechanisms that we describe here for IgE responses of helminth-infected mice can be translated to human allergic IgE responses. Most studies with allergic patients used samples from peripheral blood and reported a diverse and somatically mutated IgE repertoire similar to our murine data [43]. However, the local IgE response in mucosal tissues might be different to what can be detected in the peripheral blood. In-depth analysis of the IgE repertoire in different tissues and the overlap between the repertoire of IgE and other isotypes has not been performed. The NGS analysis we described here for the mouse can be adapted to the human immune system so that we expect to see a tremendous gain of information regarding development, distribution, and persistence of the allergic IgE response in the near future.

In conclusion, we directly demonstrate by using NGS analysis that sequential IgE switching via IgG1 dominates the primary and secondary IgE response to the helminth *N*. *brasiliensis*. The main population of memory IgE precursor cells with proliferative capacity appeared to be located in the LN and spleen rather than in the bone marrow. Furthermore, we found that the memory IgE response is affected by selective deletion of IL-4/IL-13 from T cells or deletion of IgG1^+^ B cells, while purified IgG1^+^ B cells gave rise to IgE-producing PCs upon transfer and *N*. *brasiliensis* infection of unmanipulated WT mice. These results strongly suggest that the memory IgE response which accounts for relapsing allergic disorders is driven by IgG1^+^ precursor cells. If the human allergic IgE response is subject to the same mechanisms we describe here, then therapeutic strategies should be developed to target or prevent development of allergen-specific IgG1^+^ memory B cells.

## Methods

### Ethics Statement

The animal experiments were performed in accordance with the German animal protection law and the EU guidelines 86/809. The experiments were approved by the Department of Animal Protection of the Government of Lower Franconia, Germany (license numbers 54–2532.1-23/14 and 54–2532.1-26/10).

### Mice

BALB/c, C57BL/6_Ly5.1 (B6.SJL-*Ptprc* ^*a*^ *Pepc* ^*b*^ */BoyJ*), and Rosa26-YFP reporter mice (B6.129X1-*Gt(ROSA)26Sor* ^*tm1(EYFP)Cos*^/*J*) were originally obtained from The Jackson Laboratory. *Cγ1* ^Cre/Cre^ mice [44] were crossed to Rosa26-YFP mice to generate *Cγ1* ^Cre/+^ *Rosa26* ^loxP-STOP-loxP-eYFP/+^ mice. We further used IgE^–/–^_BALB/c mice [45], IL-4/IL-13^−/−^_BALB/c (4-13ko) mice [13], CD4Cre mice [46], conditional IL-4/IL-13-deficient mice [47], Mcpt8Cre_C57BL/6 mice [35], and ΔdblGata_BALB/c mice [34]. IgE^ki/ki^_C57BL/6 mice have been described [36]. In these mice, the first three exons of the Cγ1 gene were replaced by the first four exons of the Cε gene. All mice had been backcrossed at least 9 generations to BALB/c or C57BL/6 background and used between 6 and 14 wk of age.

### Infection

Mice were infected subcutaneously at the base of the tail with 500 L3 stage larvae of *N*. *brasiliensis* as described [35].

### Flow Cytometry

Cytophilic IgE was efficiently removed by short treatment with acetate buffer as described [48]. Single cell suspensions were washed with FACS buffer (PBS, 2% FCS, 1 mg/mL NaN_3_) and incubated with anti-CD16/CD32 blocking antibody (clone 2.4G2, BioXcell, West Lebanon, NH) for 5 min at room temperature followed by staining with the following antibodies: Fluorescein isothiocyanate (FITC)- or Alexa Fluor 647 (A647)-labeled anti-CD45R (B220) (clone RA3-6B2), FITC-labeled anti-CD4 (clone RM4-5), PerCP-Cy5.5-labeled anti-CD45.2 (Ly5.2) (clone 104), A488- or A647-labeled anti-GL-7 (clone GL-7), were purchased from eBioscience (San Diego, CA); Biotin-, BV510- or FITC-labeled anti-IgE (clone R35-72), and allophycocyanin (APC)-labeled anti-CD138 (clone 281–2) were purchased from BD Biosciences (San Jose, CA); Biotin- or phycoerythrin (PE)-labeled anti-IgG1 (clone RMG1-1), pacific blue (PB)-labeled anti-CD45.1 (Ly5.1) (clone A20), PB-labeled anti-CD4 (clone RM4-5), PE-Cy7-labeled anti-CD38 (clone 90), and A647-labeled anti-CD49b (clone HMα2) were purchased from Biolegend (San Diego, CA); VioBlue-labeled B220 (clone RA3-6B2) was purchased from Miltenyi Biotec (Bergisch Gladbach, Germany). Permeabilization of cells was achieved using intracellular staining buffers from Biolegend. To detect biotinylated antibodies PE-Cy7–labeled streptavidin (BD Biosciences) or APC-eFluor780-labeled streptavidin (eBioscience) were used. Dead cells were excluded during acquisition on FACSCanto II (BD Biosciences, San Jose, CA). Sorts were performed with FACSAria II (BD Biosciences) or S3 Cell Sorter (Bio-Rad, Hercules, CA). Data were analyzed with FlowJo software (Tree Star, Ashland, OR).

### Serum ELISA

IgE levels in the serum of naive and infected mice were determined with purified anti-IgE (clone R35–72, BD Biosciences) for coating and biotinylated anti-IgE (clone R35–118, BD Biosciences) for detection. For detection of allotype-specific IgE, biotin-labeled anti-IgE^a^ (UH297) and biotin-labeled anti-IgE^b^ (JKS-6) were used (Biolegend). IgG1 ELISA was performed with a commercial ELISA kit (SouthernBiotech).

### NGS

Mice were infected subcutaneously with 500 L3 larvae *N*. *brasiliensis*, and bone marrow, lung, spleen, and LN were collected at day 15 after primary and day 9 after secondary infection. Organs or sort-purified PCs and GC B cells were homogenized in RLT buffer (Qiagen, Hilden, Germany), and RNA was isolated from lysates by RNeasy Mini kit (Qiagen). cDNA synthesis was performed with SuperScript III Reverse Transcriptase with oligo(dT)_20_ primer (both from Life Technologies, Darmstadt, Germany). To amplify the coding regions for the variable parts of the heavy chains of IgG1 and IgE, PCRs were performed with Platinum Taq DNA Polymerase (Life Technologies) and forward primer VHall 5´- cgtatcgcctccctcgcgccatcag(MID)AGGTSMARCTGCAGSAGTCWGG-3´ [29] specific for Vh gene families 1, 2, 3, 5, and 14 in combination with reverse primers binding in the Cγ1 or Cγ2a region 5´-ctatgcgccttgccagcccgctcagAGAGGTCAGACTGCAGGACAG-3´ or Cε region 5´-ctatgcgccttgccagcccgctcagTCTGAATACCAGGTCACAGTC-3´. The gene-specific primer sequences (underlined) were modified by addition of 454 adaptors, which are necessary for NGS analysis. MID (multiple identifier) specifies a 4-nt sequence used in six different variations to identify samples within single lanes. Amplicons were prepared with the GS FLX Titanium SV emPCR kit (Lib-A) for ultra-deep 454 pyrosequencing on the Genome Sequencer FLX system (Roche Diagnostics, Branford, CT) as described by the manufacturer.

For one set of samples from primary infection, PCRs were run with a forward primer VHall 5´-AGGTSMARCTGCAGSAGTCWGG-3´ in combination with reverse primers binding in the constant Cμ region 5´-ATGGTGCTGGGCAGGAAGTC-3´, Cγ1 or Cγ2a region 5´-AGAGGTCAGACTGCAGGACAG-3´, Cε region 5´-TCTGAATACCAGGTCACAGTC-3´, or Cα region 5´-ATCAGGCAGCCGATTATCAC-3´. PCR conditions were as follows: 94°C, 5 min; 35 × (94°C, 30 s; 64,5°C, 30 s; 72°C, 35 s); and 72°C, 5 min. Amplicons were purified by gel extraction with QIAquick Gel Extraction kit (Qiagen) and quantified by Quant-iT dsDNA HS Assay kit and the Qubit fluorometer (both from Life technologies). Amplicons were sequenced by Microsynth (Balgach, Switzerland) with the Genome Sequencer FLX system after 454 adaptors and MID sequences had been added.

### Sequence Analysis

Sequences were assigned to individual samples according to their MID, and sequences shorter than 320 bp and Cγ2a sequences were excluded. Sequences were further analyzed with ImMunoGeneTics (IMGT) HighV-QUEST [49,50], a web portal allowing for the analysis of high numbers of sequences. All sequences were compared against reference sequences from the IMGT database. Results obtained from IMGT were filtered for productive sequences and further analyzed with Excel (Microsoft) and VBA (Visual Basic for Applications), as previously described [28]. SHM frequencies were calculated as the number of mutations divided by the number of all nucleotides of the given frame work regions and CDRs. Morisita-Horn-indices (MHI) were calculated to compare the overlap of CDR3 sequences between different isotypes. A value of one means that sequences are identical, whereas a value of zero means that they are completely different [51]. 1,000 CDR3 sequences of each mouse and each isotype were randomly chosen and MHI was calculated with formula MHI (p_1_ p_2_) = 2 p_1_ p_2_ / (p_1_^2^ + p_1_^2^) where p1 and p2 are the two normalized populations that should be compared to each other. IMGT sequence files are accessible at the Dryad repository: http://dx.doi.org/10.5061/dryad.8bj97 [52].

### Statistics

*p*-values were calculated with unpaired two-sided Student's *t* test using SigmaPlot (Systat Software Inc., San Jose, CA). *p* < 0.05 was considered statistically significant.

## Supporting Information

S1 Data Raw data for analyses shown in the Figures and Supplemental Figures of the manuscript. (XLSX)

S1 Fig Comparison of two different staining techniques for flow cytometric analysis of IgE-expressing B cells and plasma cells (related to Fig 1). Single cell suspensions from mediastinal LN were prepared on day 12 after primary *N*. *brasiliensis* infection of wild-type BALB/c (WT) or IgE-deficient mice (IgE-ko). Cells were either incubated with excess amounts of unlabeled anti-IgE antibody to block surface IgE (surface block) or washed with acidic buffer to remove cytophilic IgE from the cell surface (acid treatment) followed by intracellular IgE staining. (A) Samples are gated on GC B cells (B220^+^CD38^−^GL-7^+^ as shown in S9 Fig) and display IgD versus IgE. (B) Samples are gated on plasma cells (B220^lo^CD138^+^ as shown in S9 Fig) and display c-Kit versus IgE.
(TIF)

S2 Fig Ig repertoire analysis in OVA/alum-immunized mice (related to Fig 1). BALB/c mice were immunized intraperitoneally (i.p.) with OVA/alum on day 0 and day 7, challenged intranasally on days 13 and 14 before Ig repertoires were analyzed on day 15 by NGS. (A) Number of different CDR3 sequences among 1,000 randomly selected sequences from IgE, IgG1, and IgM pools. (B) Heat maps demonstrate that the most abundant CDR3 sequences in the IgE repertoires of each mouse are often shared with the IgG1 but not the IgM repertoire. The brightest green means that this CDR3 sequence was found in at least 0.5% of all sequences. (C) Morisita-Horn indices as a measure for the relatedness between 1,000 randomly picked sequences of the IgG1 and IgE repertoires or the IgM and IgE repertoires. (D) Number of somatic mutations in the VH genes of IgG1 and IgE. (E) Distribution of somatic mutations over indicated regions of the VH genes. Bars show the mean + SEM from three mice.
(TIF)

S3 Fig Ig repertoire analysis in mesenteric LN of *N*. *brasiliensis*-infected mice (related to Fig 2). Five BALB/c mice were infected with *N*. *brasiliensis*, and Ig repertoires were analyzed on day 15 after infection. (A) Repertoire diversity displayed as mean + SD different sequences in 1,000 randomly chosen sequences. (B) Relative usage of different V_H_, D_H_, and J_H_ segments among indicated isotypes. Bars show the mean + SD from five mice. (C) Heat maps show the overlap between the first 50 most frequent CDR3 sequences in the IgE repertoire with the same CDR3 sequences in the IgG1 and IgM repertoires from five individual mice. Each row indicates one unique CDR3 sequence ordered by decreasing frequency in the IgE pools. The brightest green indicates CDR3 sequences with an abundance of ≥ 1%. Bar graph shows the Morisita-Horn Index for the relatedness between the IgE and IgG1 repertoires and the relatedness between the IgE and IgM repertoires based on 1,000 randomly chosen sequences from each isotype. (D) Same analysis as in (C), but IgA was used instead of IgE. (E) Direct comparison of the IgE and IgA repertoires. ** *p* < 0.01 by Student’s *t* test.
(TIF)

S4 Fig Usage of V_H_, D_H_, and J_H_ segments after primary *N*. *brasiliensis* infection (related to Fig 6). Two individual mice were analyzed at day 15 after primary *N*. *brasiliensis* infection for usage of indicated V_H_, D_H_, and J_H_ segments among 1,000 randomly chosen IgE and IgG1 sequences from bone marrow (BM), lung, spleen, and mesenteric LN.
(TIF)

S5 Fig Usage of V_H_, D_H_, and J_H_ segments after secondary *N*. *brasiliensis* infection (related to Fig 6). Two individual mice were analyzed at day 9 after secondary *N*. *brasiliensis* infection for usage of indicated V_H_, D_H_, and J_H_ segments among 1,000 randomly chosen IgE and IgG1 sequences from bone marrow (BM), lung, spleen, and mesenteric LN.
(TIF)

S6 Fig *N*. *brasiliensis* memory B cells have a competitive advantage over naïve B cells (related to Fig 7). (A) Outline of transfer experiment referring to data in B–D. IgH^b^/Ly5.1 mice were infected with *N*. *brasiliensis* 4 wk before cell transfer to establish memory mice. Cell suspension from SP or LN from memory IgH^b^/Ly5.1 and naïve IgH^a^/Ly5.2 mice were mixed at a 1:1 ratio of B cells from each mouse and transferred into Rag1^–/–^mice. Mesenteric LN and serum were analyzed 12 d after *N*. *brasiliensis* infection of Rag1^−/−^ recipient mice. (B) Representative plots showing transferred CD4^+^ T cells and B220^+^ B cells (left) and percentage of naïve (Ly5.2^+^) and memory (Ly5.1^+^) CD4^+^ T cells (middle plot) or B220^+^ B cells (right plot). (C) Bar graph shows the percentage of B cells from naïve or memory donor cells from LN and spleen (SP) in the mesenteric LN of infected Rag1^−/−^ recipient mice. (D) Frequency of Ly5.1^+^ and Ly5.2^+^ B cells within the CD38^+^IgD^+^ gate (mainly naïve B cells) and CD38^+^IgD^−^ gate (mainly memory B cells). Dot plots are gated from the parental gate shown in S13 Fig. (E) Bar graph shows IgE produced by B cells from memory mice (detected as IgE^b^) or B cells from naive mice (detected as IgE^a^) in the serum of infected Rag1^–/–^recipient mice. Bars in (C) and (E) show the mean + SD from four mice per group.
(TIF)

S7 Fig Sorting gate to isolate the B cell and PC populations used for transfers in Fig 9E (related to Fig 9E). The indicated sorting gates were used to purify IgG1-expressing B cells and IgG1-negative PCs (upper part) or to remove IgM-, IgD-, and IgG1-expressing B cells or IgM-, IgD-, and IgE-expressing B cells (lower part) in order to transfer enriched and untouched IgE- or IgG1-expressing B cells for the experiment shown in Fig 9E.
(TIF)

S8 Fig Gating strategy to get to the parental gates from LN samples analyzed in the related figures (related to Fig 1B and 1C, Fig 9A and 9B, S6B Fig). (TIF)

S9 Fig Gating strategy to get to the parental gates for GC B cells and PCs from LN samples analyzed in the related figures (related to Fig 1D, Fig 4B–4D and 4F and S1 Fig). (TIF)

S10 Fig Gating strategy to get to the parental gates of the dot plots shown in Fig 5A to determine the frequency of PCs in different tissues (related to Fig 5A). (TIF)

S11 Fig Expression of IgE and IgG1 in PCs gated as indicated in Fig 5A and S10 Fig (related to Fig 5B–5E). (TIF)

S12 Fig Gating strategy to identify the B cell populations in Fig 8 (related to Fig 8B and 8C). (A) Gating strategy to distinguish transferred Ly5.1 and Ly5.2 B cells. (B) Gating strategy to analyze CD38 expression on Ly5.2+ and Ly5.2- B cells.
(TIF)

S13 Fig Gating strategy for the parental gates used for analysis in S6D Fig (related to S6D Fig). (TIF)

## Abbreviations

BCR: B cell receptor
CSR: class switch recombination
GC: germinal center
GFP: green fluorescent protein
ILC2: type 2 innate lymphoid cell
LN: lymph nodes
NGS: next generation sequencing
NKT: natural killer T cells
PCs: plasma cells
PCR: polymerase chain reaction
RT-PCR: reverse transcription polymerase chain reaction
SD: standard deviation
SEM: standard error of the mean
SHM: somatic hypermutations
T_FH_: follicular T helper
VDJ: variable, diversity, and joining
WT: wild-type
