# Supplementary figures and images for: The Extracellular Domains of IgG1 and T Cell-Derived IL-4/IL-13 Are Critical for the Polyclonal Memory IgE Response In Vivo
Source: PLoS Biol. 2015 Nov 2;13(11):e1002290. doi: 10.1371/journal.pbio.1002290 (PMC4629909; doi:10.1371/journal.pbio.1002290)

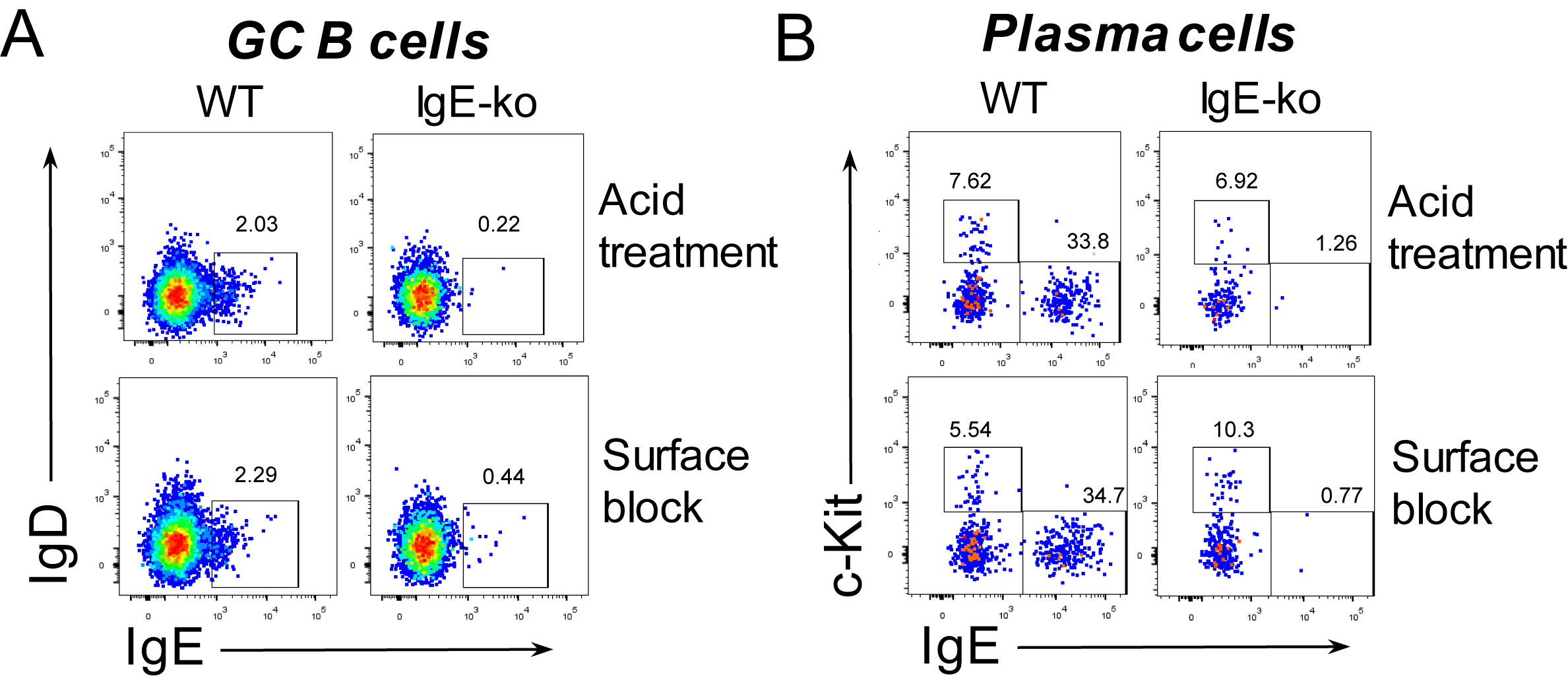

Supplement: S1 Fig — Single cell suspensions from mediastinal LN were prepared on day 12 after primary N. brasiliensis infection of wild-type BALB/c (WT) or IgE-deficient mice (IgE-ko). Cells were either incubated with excess amounts of unlabeled anti-IgE antibody to block surface IgE (surface block) or washed with acidic buffer to remove cytophilic IgE from the cell surface (acid treatment) followed by intracellular IgE staining. (A) Samples are gated on GC B cells (B220+CD38−GL-7+ as shown in S9 Fig) and display IgD versus IgE. (B) Samples are gated on plasma cells (B220loCD138+ as shown in S9 Fig) and display c-Kit versus IgE. (TIF) [file pbio.1002290.s002.tif]

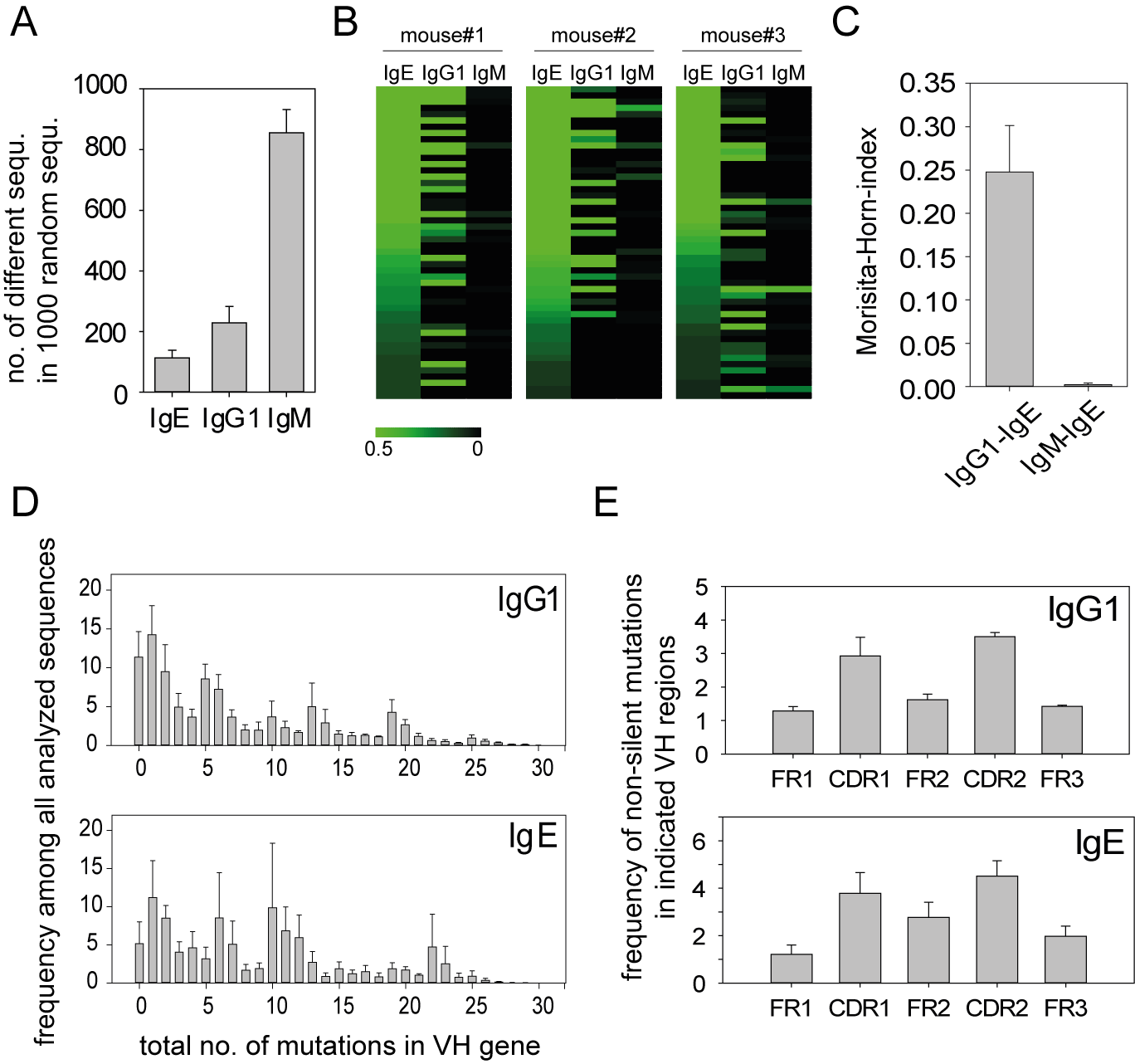

Supplement: S2 Fig — BALB/c mice were immunized intraperitoneally (i.p.) with OVA/alum on day 0 and day 7, challenged intranasally on days 13 and 14 before Ig repertoires were analyzed on day 15 by NGS. (A) Number of different CDR3 sequences among 1,000 randomly selected sequences from IgE, IgG1, and IgM pools. (B) Heat maps demonstrate that the most abundant CDR3 sequences in the IgE repertoires of each mouse are often shared with the IgG1 but not the IgM repertoire. The brightest green means that this CDR3 sequence was found in at least 0.5% of all sequences. (C) Morisita-Horn indices as a measure for the relatedness between 1,000 randomly picked sequences of the IgG1 and IgE repertoires or the IgM and IgE repertoires. (D) Number of somatic mutations in the VH genes of IgG1 and IgE. (E) Distribution of somatic mutations over indicated regions of the VH genes. Bars show the mean + SEM from three mice. (TIF) [file pbio.1002290.s003.tif]

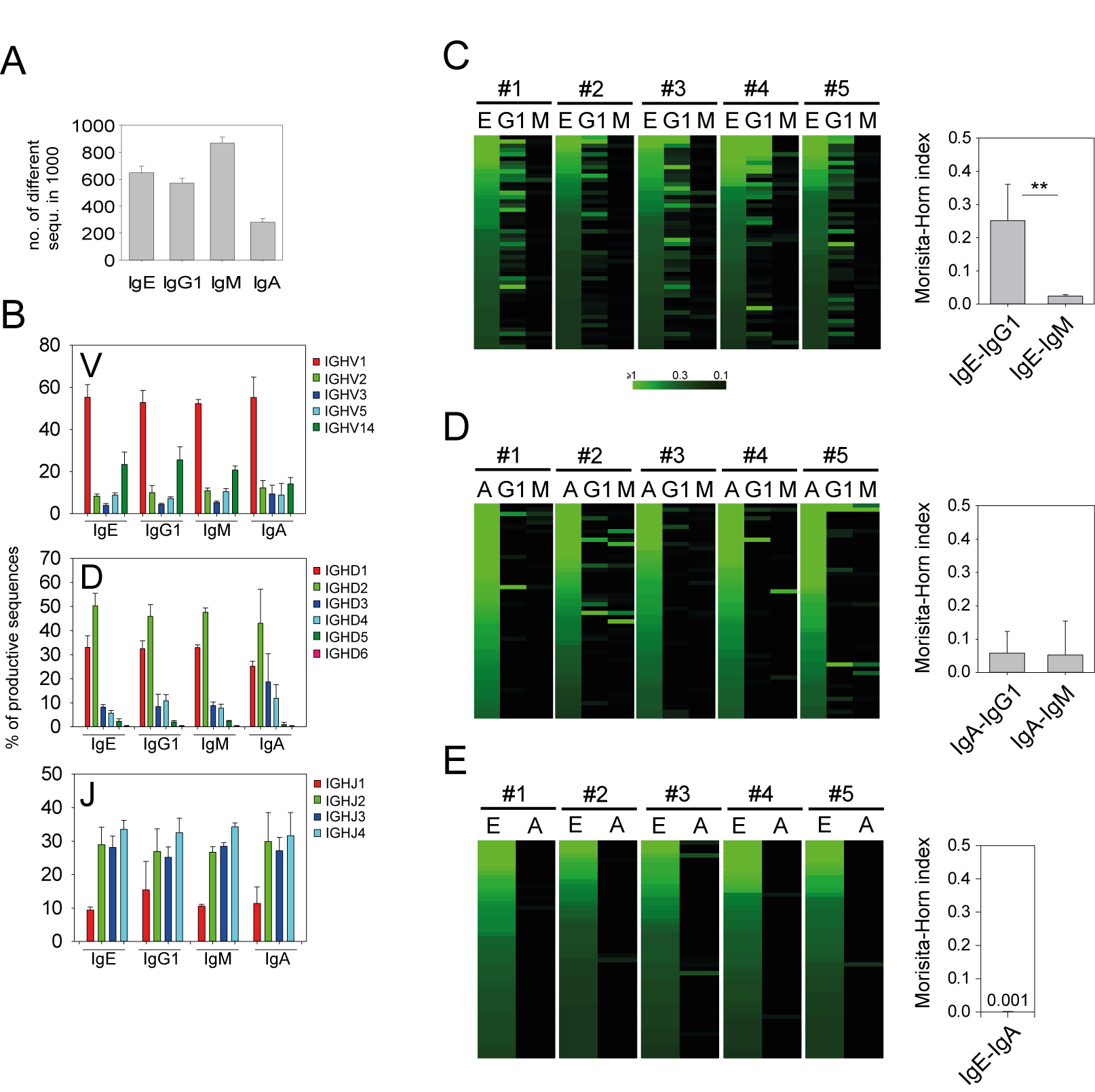

Supplement: S3 Fig — Five BALB/c mice were infected with N. brasiliensis, and Ig repertoires were analyzed on day 15 after infection. (A) Repertoire diversity displayed as mean + SD different sequences in 1,000 randomly chosen sequences. (B) Relative usage of different VH, DH, and JH segments among indicated isotypes. Bars show the mean + SD from five mice. (C) Heat maps show the overlap between the first 50 most frequent CDR3 sequences in the IgE repertoire with the same CDR3 sequences in the IgG1 and IgM repertoires from five individual mice. Each row indicates one unique CDR3 sequence ordered by decreasing frequency in the IgE pools. The brightest green indicates CDR3 sequences with an abundance of ≥ 1%. Bar graph shows the Morisita-Horn Index for the relatedness between the IgE and IgG1 repertoires and the relatedness between the IgE and IgM repertoires based on 1,000 randomly chosen sequences from each isotype. (D) Same analysis as in (C), but IgA was used instead of IgE. (E) Direct comparison of the IgE and IgA repertoires. ** p < 0.01 by Student’s t test. (TIF) [file pbio.1002290.s004.tif]

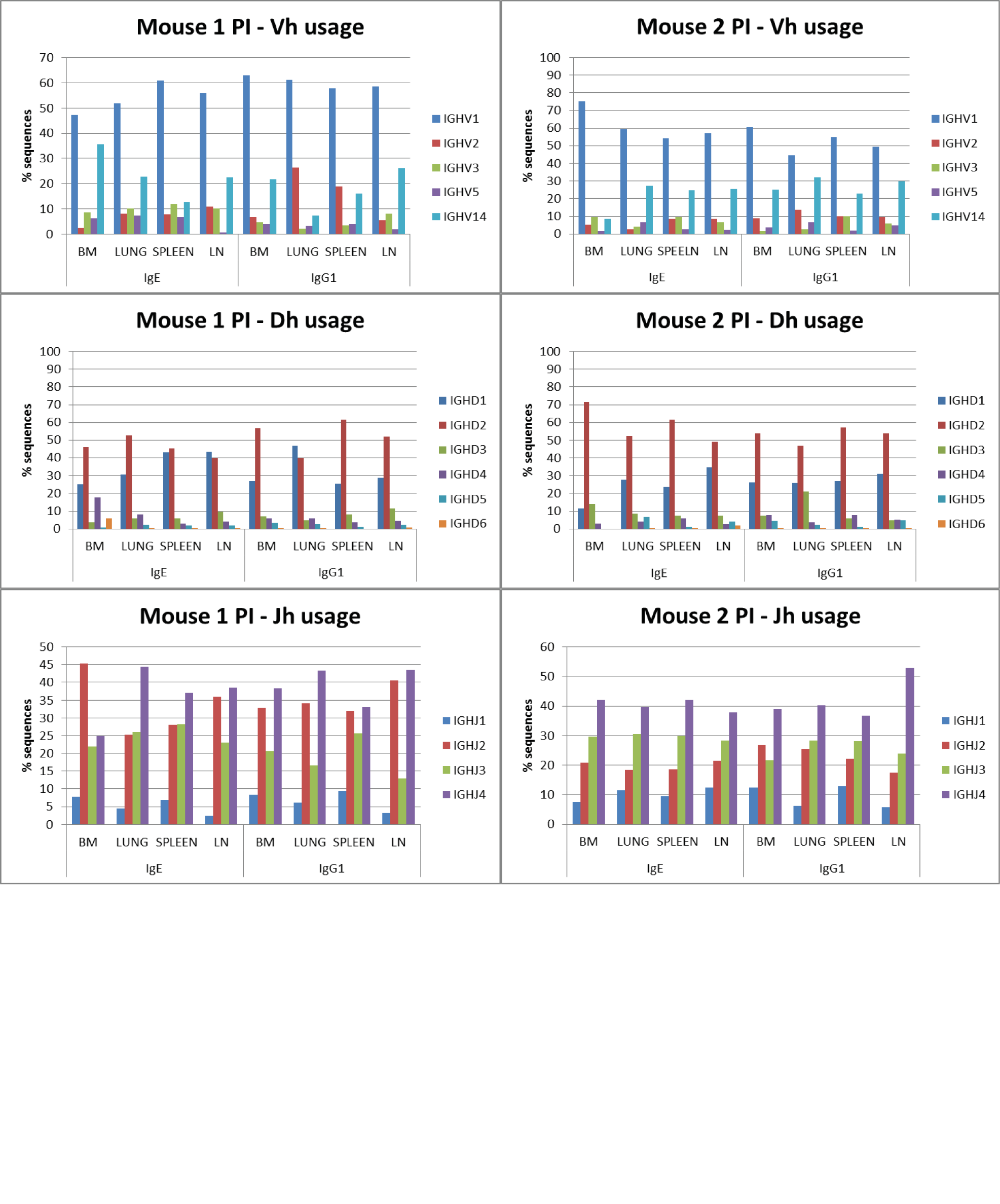

Supplement: S4 Fig — Two individual mice were analyzed at day 15 after primary N. brasiliensis infection for usage of indicated VH, DH, and JH segments among 1,000 randomly chosen IgE and IgG1 sequences from bone marrow (BM), lung, spleen, and mesenteric LN. (TIF) [file pbio.1002290.s005.tif]

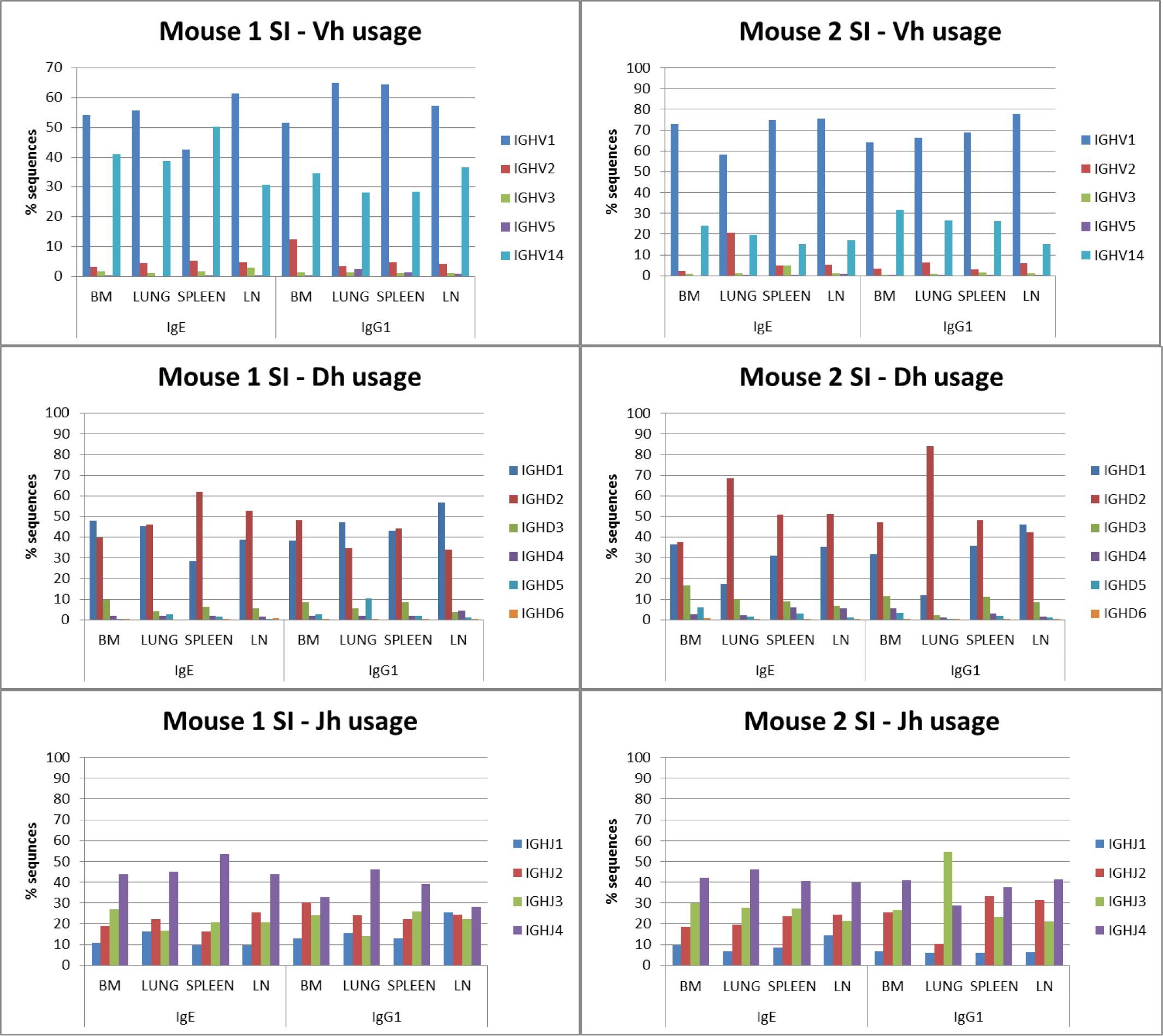

Supplement: S5 Fig — Two individual mice were analyzed at day 9 after secondary N. brasiliensis infection for usage of indicated VH, DH, and JH segments among 1,000 randomly chosen IgE and IgG1 sequences from bone marrow (BM), lung, spleen, and mesenteric LN. (TIF) [file pbio.1002290.s006.tif]

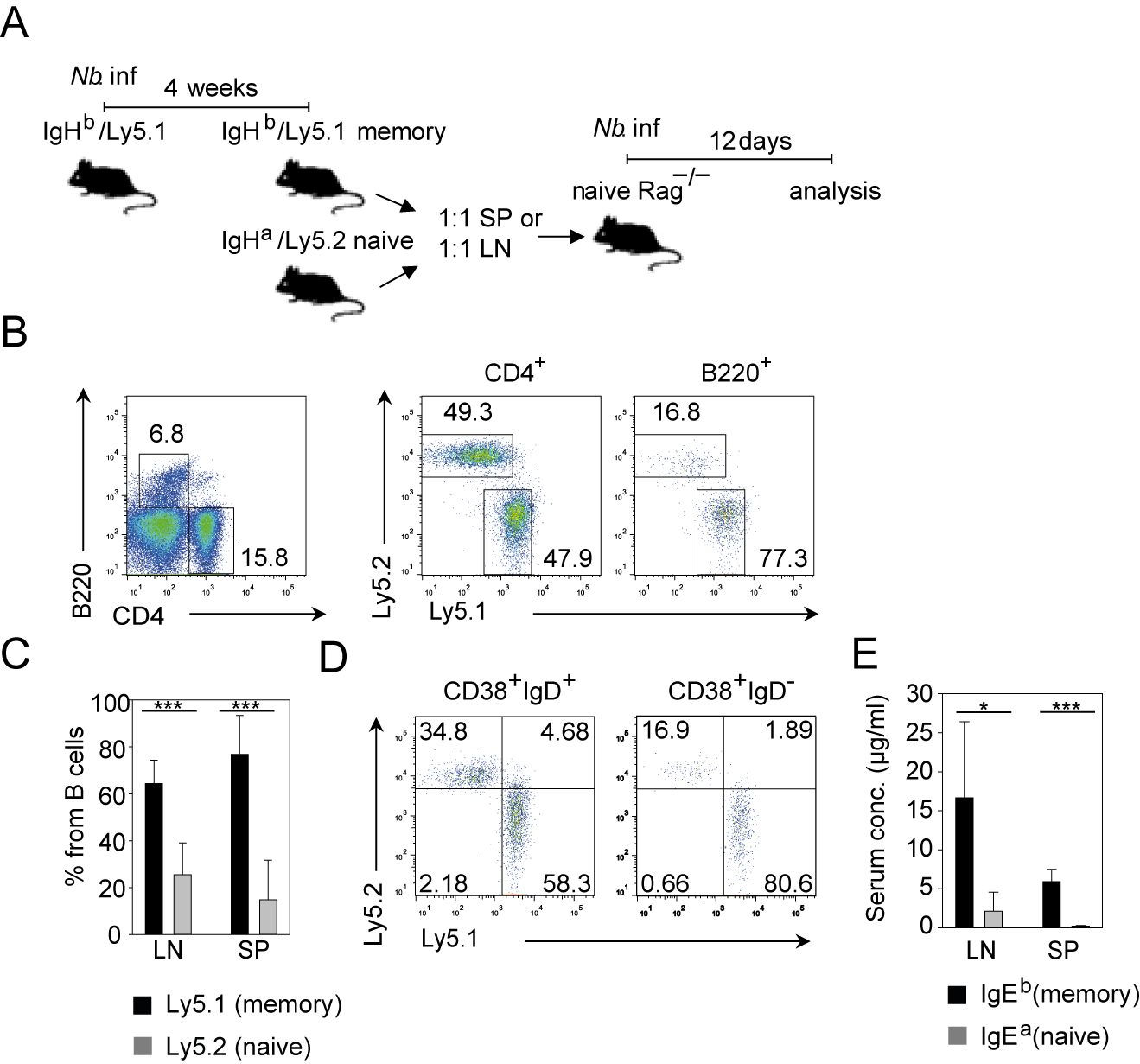

Supplement: S6 Fig — (A) Outline of transfer experiment referring to data in B–D. IgHb/Ly5.1 mice were infected with N. brasiliensis 4 wk before cell transfer to establish memory mice. Cell suspension from SP or LN from memory IgHb/Ly5.1 and naïve IgHa/Ly5.2 mice were mixed at a 1:1 ratio of B cells from each mouse and transferred into Rag1–/–mice. Mesenteric LN and serum were analyzed 12 d after N. brasiliensis infection of Rag1−/− recipient mice. (B) Representative plots showing transferred CD4+ T cells and B220+ B cells (left) and percentage of naïve (Ly5.2+) and memory (Ly5.1+) CD4+ T cells (middle plot) or B220+ B cells (right plot). (C) Bar graph shows the percentage of B cells from naïve or memory donor cells from LN and spleen (SP) in the mesenteric LN of infected Rag1−/− recipient mice. (D) Frequency of Ly5.1+ and Ly5.2+ B cells within the CD38+IgD+ gate (mainly naïve B cells) and CD38+IgD− gate (mainly memory B cells). Dot plots are gated from the parental gate shown in S13 Fig. (E) Bar graph shows IgE produced by B cells from memory mice (detected as IgEb) or B cells from naive mice (detected as IgEa) in the serum of infected Rag1–/–recipient mice. Bars in (C) and (E) show the mean + SD from four mice per group. (TIF) [file pbio.1002290.s007.tif]

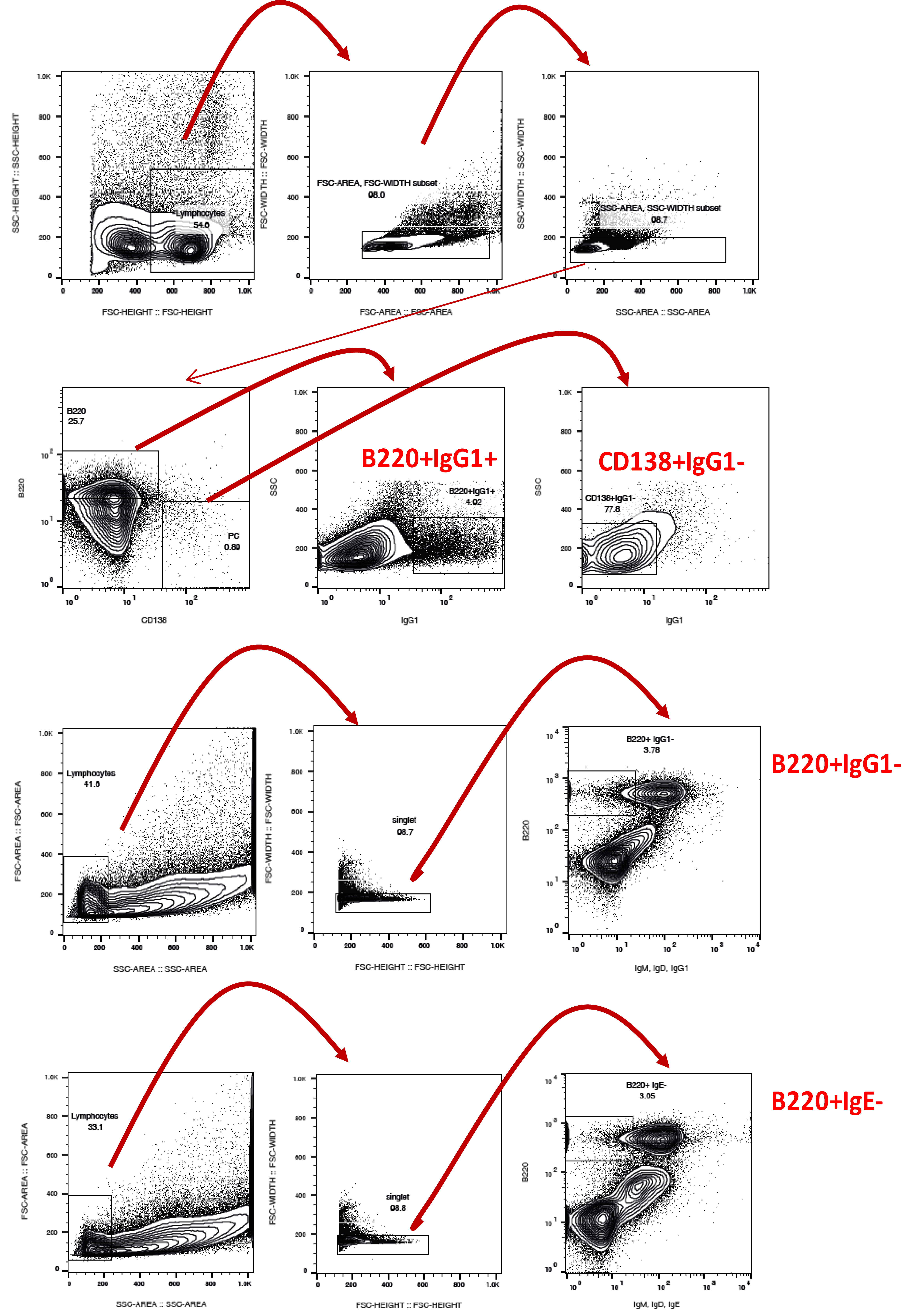

Supplement: S7 Fig — The indicated sorting gates were used to purify IgG1-expressing B cells and IgG1-negative PCs (upper part) or to remove IgM-, IgD-, and IgG1-expressing B cells or IgM-, IgD-, and IgE-expressing B cells (lower part) in order to transfer enriched and untouched IgE- or IgG1-expressing B cells for the experiment shown in Fig 9E. (TIF) [file pbio.1002290.s008.tif]

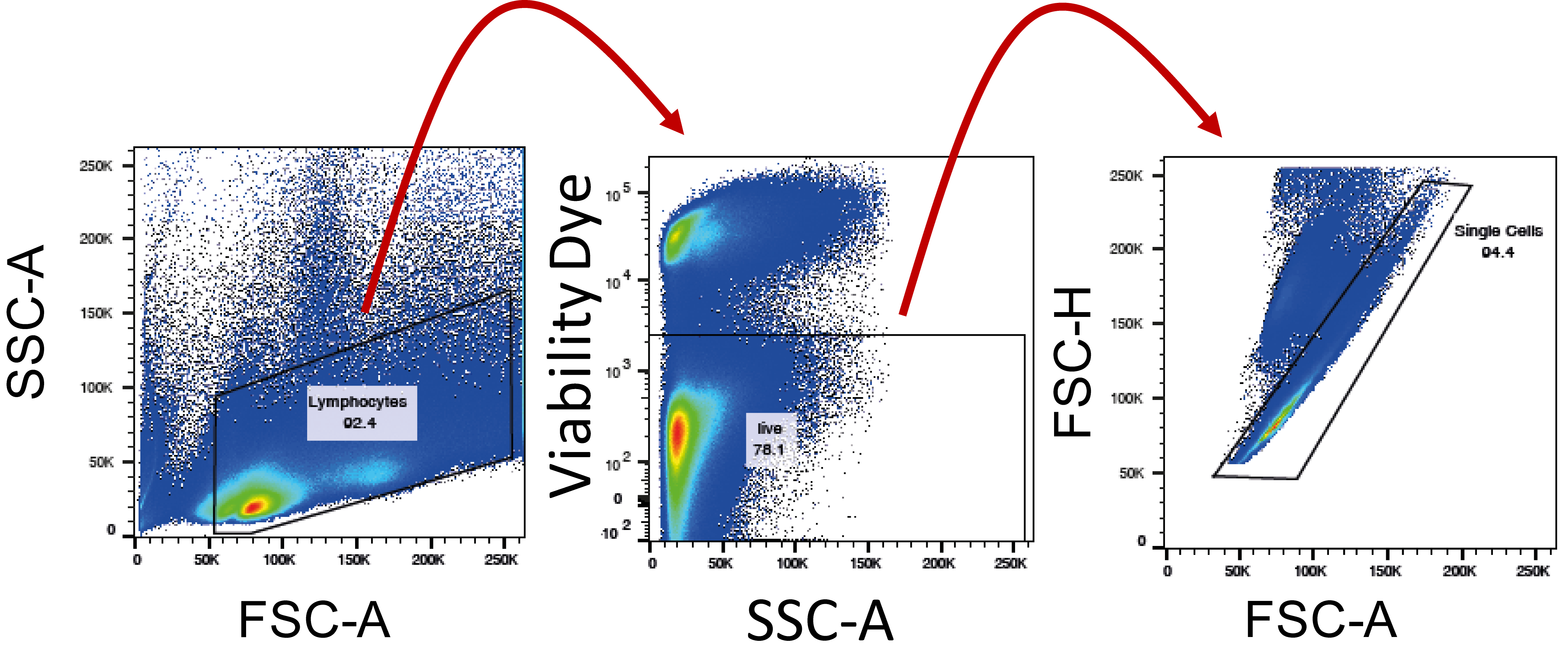

Supplement: S8 Fig — (TIF) [file pbio.1002290.s009.tif]

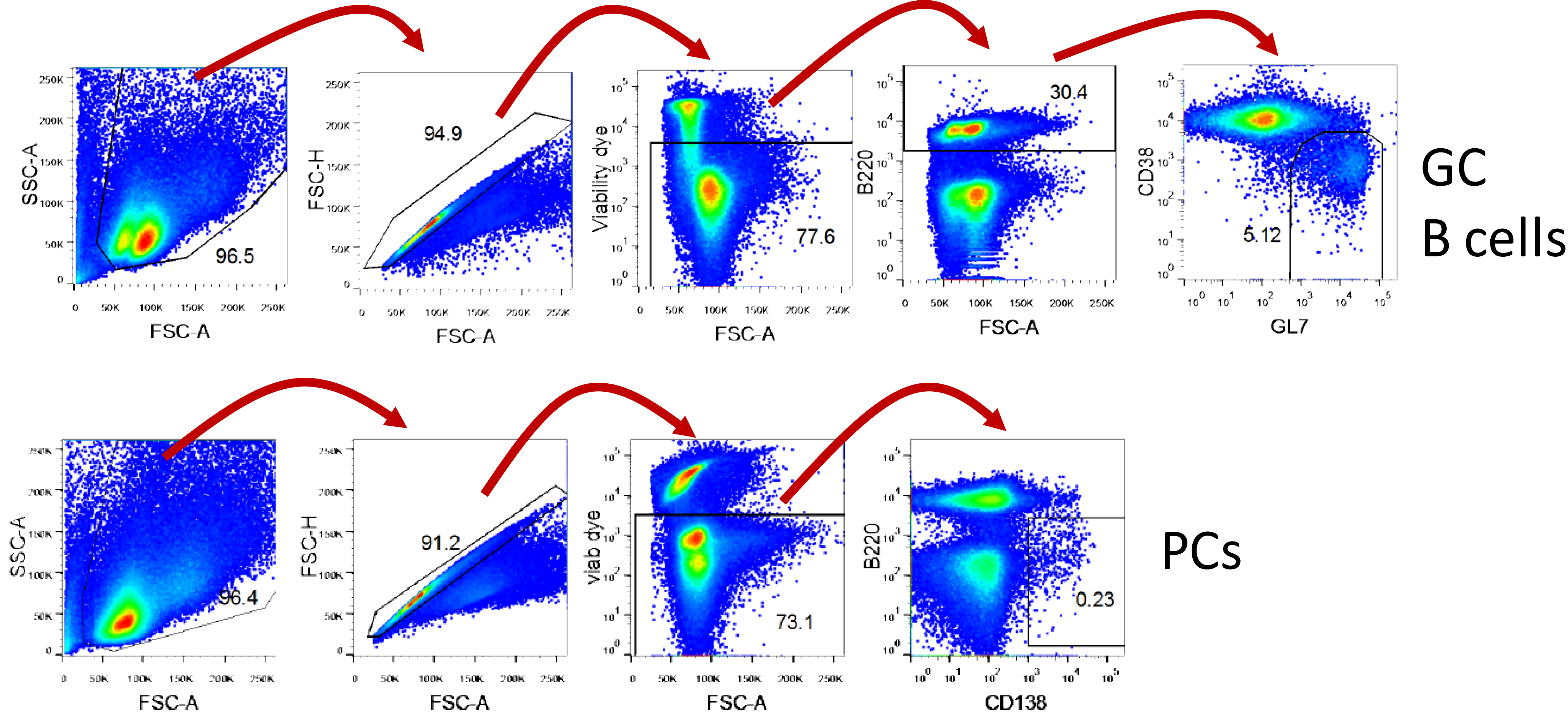

Supplement: S9 Fig — (TIF) [file pbio.1002290.s010.tif]

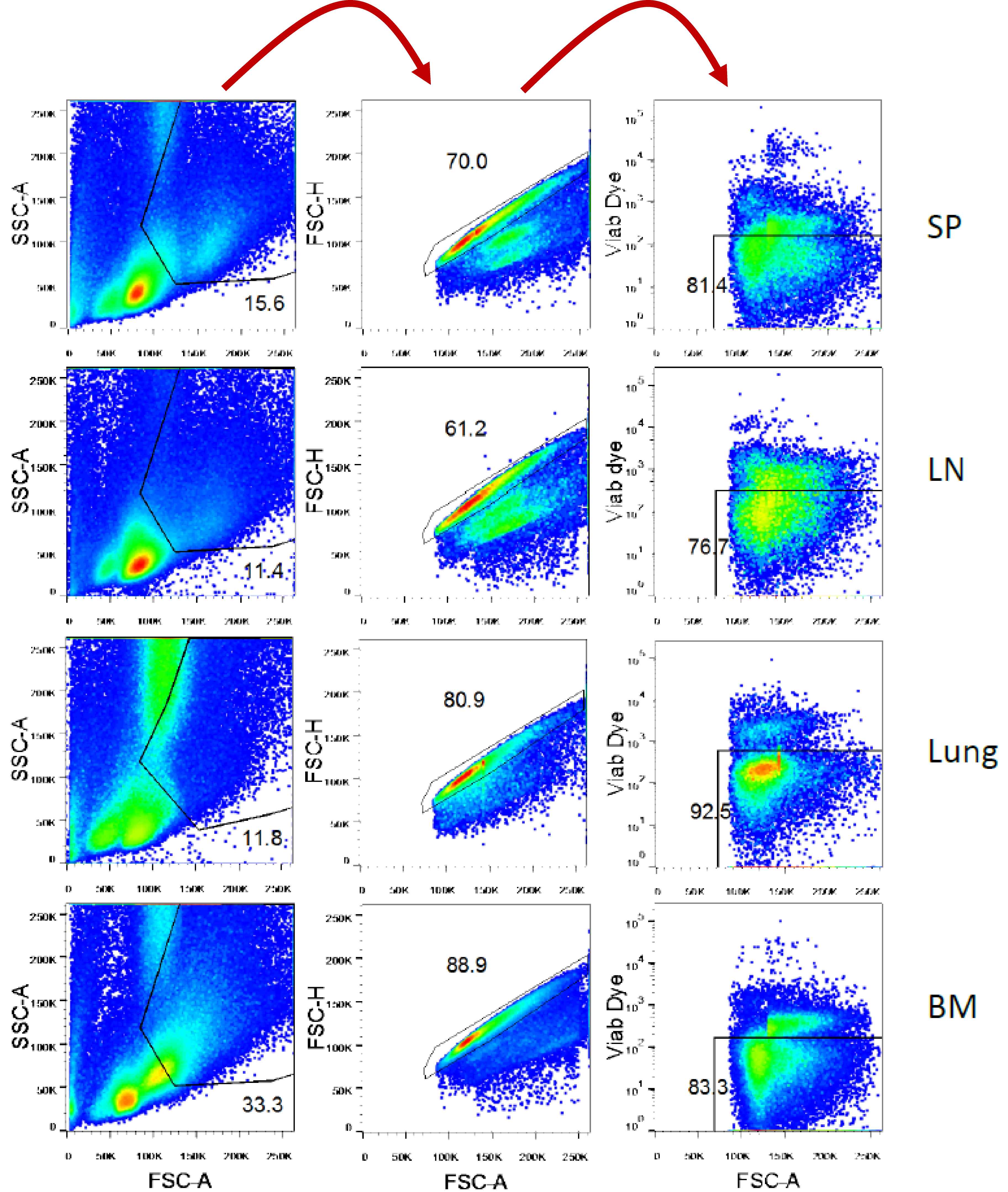

Supplement: S10 Fig — (TIF) [file pbio.1002290.s011.tif]

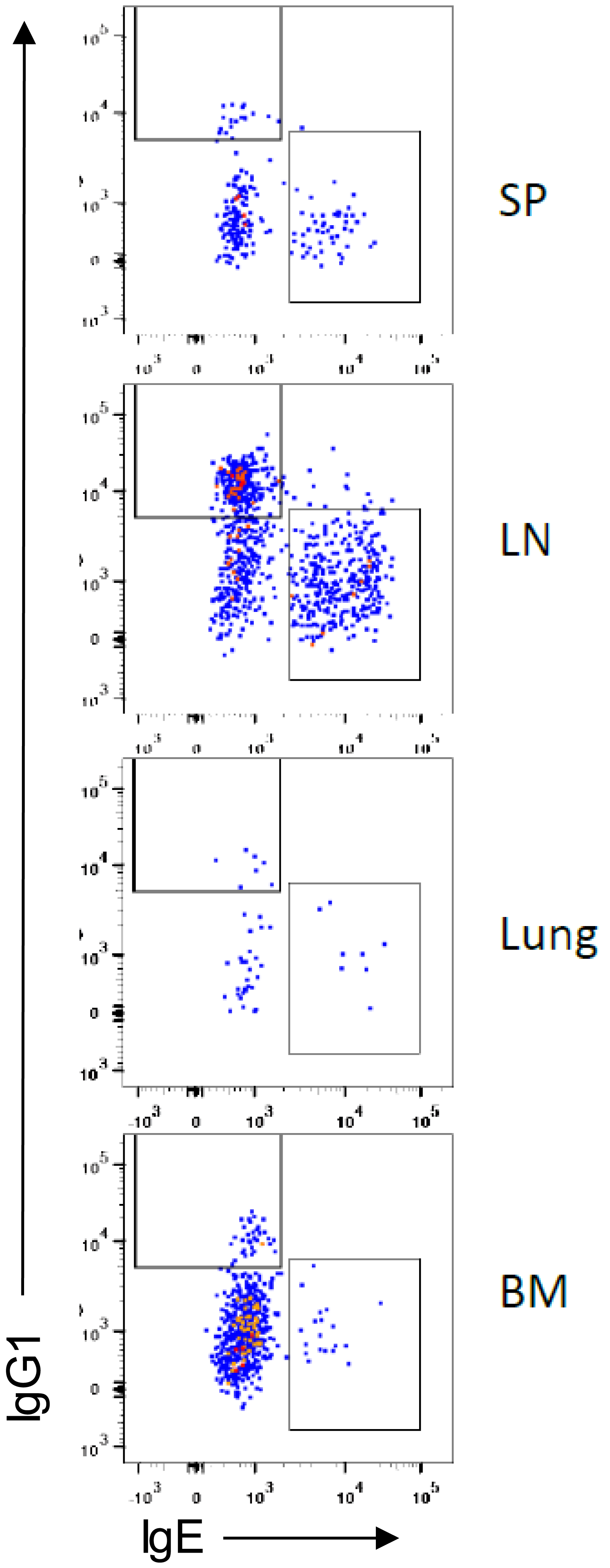

Supplement: S11 Fig — (TIF) [file pbio.1002290.s012.tif]

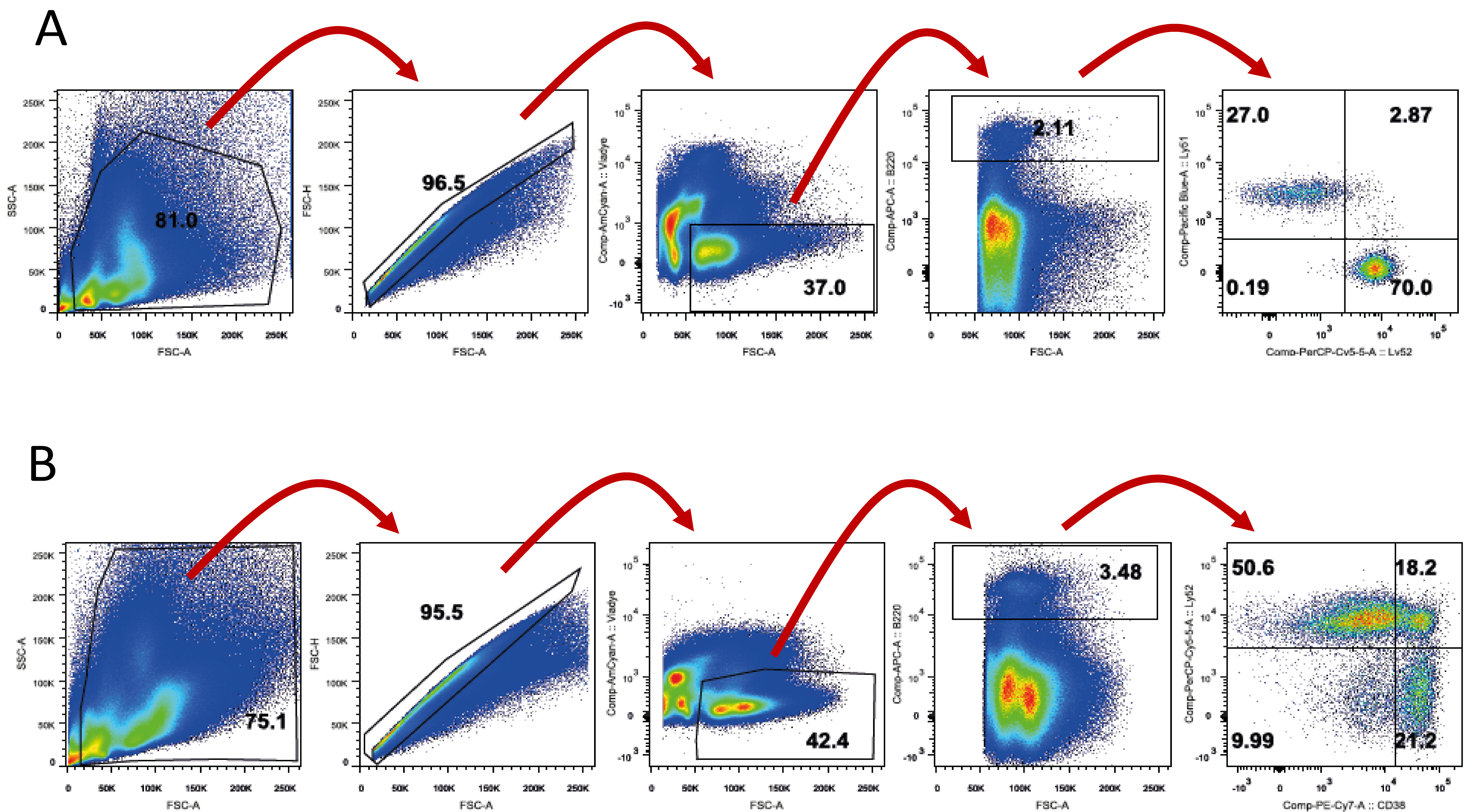

Supplement: S12 Fig — (A) Gating strategy to distinguish transferred Ly5.1 and Ly5.2 B cells. (B) Gating strategy to analyze CD38 expression on Ly5.2+ and Ly5.2- B cells. (TIF) [file pbio.1002290.s013.tif]

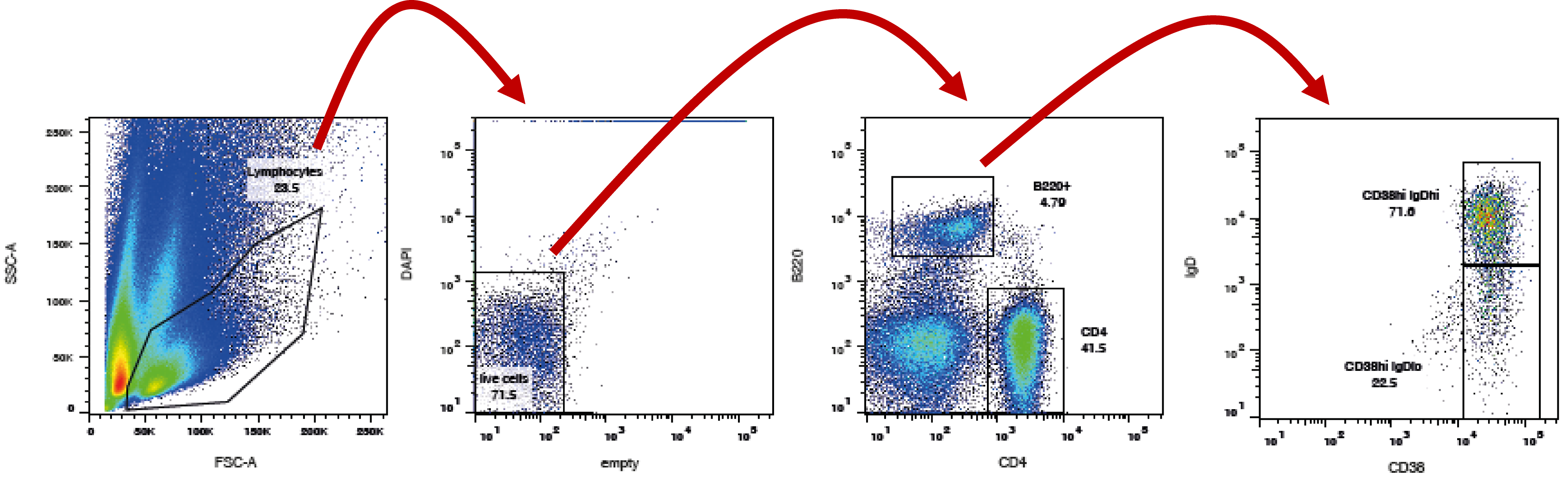

Supplement: S13 Fig — (TIF) [file pbio.1002290.s014.tif]
